# Unpatterned Bioactive Poly(Butylene 1,4-Cyclohexanedicarboxylate)-Based Film Fast Induced Neuronal-Like Differentiation of Human Bone Marrow-Mesenchymal Stem Cells

**DOI:** 10.3390/ijms21239274

**Published:** 2020-12-04

**Authors:** Francesco Morena, Chiara Argentati, Michelina Soccio, Ilaria Bicchi, Francesca Luzi, Luigi Torre, Andrea Munari, Carla Emiliani, Matteo Gigli, Nadia Lotti, Ilaria Armentano, Sabata Martino

**Affiliations:** 1 Department of Chemistry, Biology and Biotechnology, University of Perugia, 06123 Perugia, Italy; francesco.morena@unipg.it (F.M.); chiara.argentati89@gmail.com (C.A.); ilabi1983@gmail.com (I.B.); carla.emiliani@unipg.it (C.E.); 2 Department of Civil, Chemical, Environmental, and Materials Engineering–DICAM, University of Bologna, 40136 Bologna, Italy; m.soccio@unibo.it (M.S.); andrea.munari@unibo.it (A.M.); 3 Civil and Environmental Engineering Department, UdR INSTM, University of Perugia, 05100 Terni, Italy; francesca.luzi@unipg.it (F.L.); luigi.torre@unipg.it (L.T.); 4 CEMIN, University of Perugia, 06123 Perugia, Italy; 5 Department of Molecular Sciences and Nanosystems, Ca’Foscari University of Venice, 30170 Venezia Mestre, Italy; matteo.gigli@unive.it; 6 Department of Economics, Engineering, Society and Business Organization (DEIM), University of Tuscia, 01100 Viterbo, Italy

**Keywords:** stem cell-biomaterial interaction, stem cell commitment, compression moulding, solvent-casting, surface property, wettability, microstructure

## Abstract

Herein, we present poly(butylene 1,4-cyclohexanedicarboxylate) (PBCE) films characterized by an unpatterned microstructure and a specific hydrophobicity, capable of boosting a drastic cytoskeleton architecture remodeling, culminating with the neuronal-like differentiation of human bone marrow-mesenchymal stem cells (hBM-MSCs). We have used two different filming procedures to prepare the films, solvent casting (PBCE) and compression-moulding (PBCE*). PBCE film had a rough and porous surface with spherulite-like aggregations (Ø = 10–20 μm) and was characterized by a water contact angle = 100°. PBCE* showed a smooth and continuous surface without voids and visible spherulite-like aggregations and was more hydrophobic (WCA = 110°). Both surface characteristics were modulated through the copolymerization of different amounts of ether-oxygen-containing co-units into PBCE chemical structure. We showed that only the surface characteristics of PBCE-solvent-casted films steered hBM-MSCs toward a neuronal-like differentiation. hBM-MSCs lost their canonical mesenchymal morphology, acquired a neuronal polarized shape with a long cell protrusion (≥150 μm), expressed neuron-specific class III β-tubulin and microtubule-associated protein 2 neuronal markers, while nestin, a marker of uncommitted stem cells, was drastically silenced. These events were observed as early as 2-days after cell seeding. Of note, the phenomenon was totally absent on PBCE* film, as hBM-MSCs maintained the mesenchymal shape and behavior and did not express neuronal/glial markers.

## 1. Introduction

The success of tissue engineering approaches depends on the cross-talk established between stem cells and the biomaterial’s surface [1,2]. Here, the chemico-physical cues of biomaterials act as biochemical signaling that stem cells perceive and transduce in biological functions such as adhesion, proliferation, shape, and differentiation [3,4,5,6,7]. At the same time, stem cells act on biomaterials recreating their microenvironment [3]. This interplay recapitulates the dynamic physiological cross-talk between stem cells and the extracellular matrix (ECM), by which the ECM orchestrates the cell shape and functioning by its inherent topographical/chemical/physical characteristics, while stem cells change the ECM molecular composition and remodel its architecture [8,9]. In accordance with the cell tensegrity theory, ECM chemico-physical cues generate a circuit of signals that are collected by cell membrane proteins (e.g., integrins, focal adhesion proteins) and propagated (mechanotransduced) across the cytoskeleton fibers to the chromatin where a specific cell function is activated [10,11,12,13,14,15,16,17]. Similarly, biomaterials may be able to recapitulate the abovementioned mechanotransduction process dictating a specific stem cell fate decision [5,6,7,17]. It has been shown that the chemical composition, the microstructure of biomaterials (film or scaffold) and, mainly, their surface characteristics play a key role in these molecular events [18,19,20,21,22,23]. Specifically, the role of the topographical patterned surface has been associated with the induction of stem cell differentiation [24,25,26,27], the stiffness was correlated with the effect of physical forces on steering the stem cells’ fate [7,10,12,14,21,28,29], while surface wettability (different hydrophobicity/hydrophilicity ratio) modulates the cell-biomaterial interactions [22,30,31,32,33]. Generally, the surface properties work in synergy, therefore the biological effect is the result of the combination of two or more characteristics (e.g., patterned/unpatterned micro/nanostructure with smooth/rough morphology, stiffness, elasticity, and wettability) [3,18,19,20,21,22,23]. Therefore, there is a great need to elucidate these molecular events from the perspective of developing innovative and effective biomaterials for specific biomedical applications.

Within this framework, for several years now we have been developing new biomaterials acting on their chemical structure (through different strategies, such as copolymer, blend, nanocomposite), type (films, scaffold) and surface properties (flat, nanostructured, and nanopatterned films) [34,35,36,37,38,39,40,41], to investigate the mechanotransduction events that are activated at the molecular interface between stem cells and biomaterials [3,17,21,22,42,43,44]. In particular, we are interested in studying the effects of these molecular interactions on the architecture of the cells’ cytoskeleton, and in understanding how changes of the cell tensegrity influence the stem cells’ fate [14,17].

In this work, we have focused our attention on a cycloaliphatic polyester, poly(butylene 1,4-cyclohexanedicarboxylate) (PBCE), and on two of its random copolyesters containing ether-linkages, which were utilized to prepare films employing different processing techniques. The effect of surface chemico-physical cues on the fate of adult human bone marrow-mesenchymal stem cells (hBM-MSCs) have been deeply investigated. We took advantage of the peculiarity of this polymer to generate films with different surface characteristics in terms of microstructure and hydrophobicity degree based on the processing technique used: solvent casting (PBCE) and compression-moulding (PBCE*). Moreover, in solvent casted films, both surface characteristics were further finely modulated by the introduction of increasing amounts of butylene diglycolate (BDG) co-units in PBCE backbone through copolymerization [37,45]. Two random copolymers with different compositions have been prepared, i.e., BDG10 and BDG30, containing 10 and 30 mol% BDG, respectively. To establish more properly the correlation between surface characteristics and stem cells, the study was conducted by culturing the biohybrid system in growth culture medium without additional bioactive molecules and, for comparison, in growth culture medium fetal bovine serum (FBS)-free.

We have chosen hBM-MSCs for this study due to the relatively easy isolation procedure from the bone marrow and the well-established protocols for their in-vitro expansion and differentiation [46,47,48,49]. Moreover, the multipotential properties of hBM-MSCs to give rise to non-hematopoietic cell lineages, such as fibroblast-, osteogenic-, adipogenic-, chondrogenic- and neural-lineages [46,47,48,49], together with their immunomodulatory activity [50,51], make these stem cells suitable for regenerative medicine applications [46,47,52] and stem cell basic research [46,47,48,49].

We have demonstrated that the tailored combination of unpatterned microstructure and hydrophobicity degree of solvent-casted PBCE steer the hBM-MSCs fate toward the neuronal-like differentiation.

## 2. Results

### 2.1. PBCE* and PBCE, BDG10, BDG30 Polymer Films Characterization

**Figure 1:**
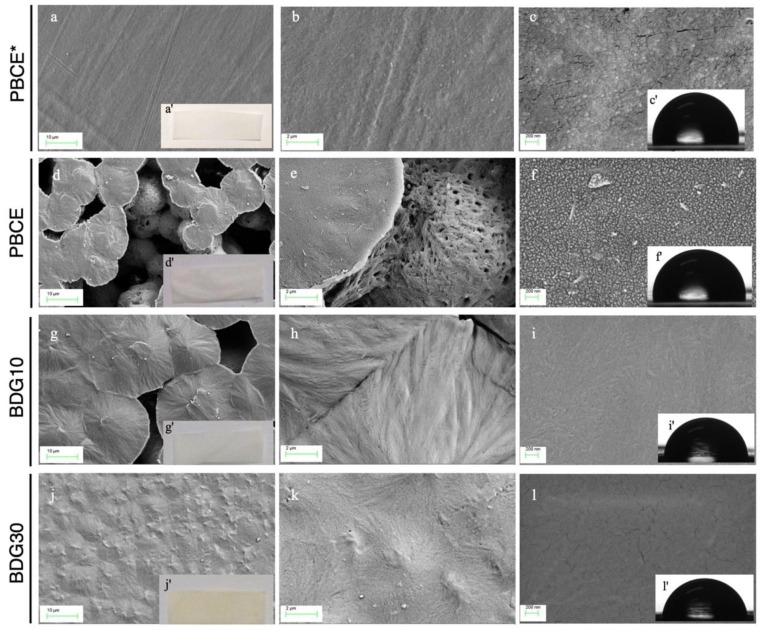
PBCE*, PBCE, BDG10 and BDG30 polymer films. Representative FESEM images of PBCE* (**a**–**c**), PBCE (**d**–**f**), BDG10 (**g**–**i**) and BDG30 (**j**–**l**) surface. The insets show optical photographs (**a^I^**,**d^I^**,**g^I^**,**j^I^**) and water contact angle images (**c^I^**,**f^I^**,**i^I^**,**l^I^**). Scale bar: (**a**,**d**,**g**,**j**): 10 μm; (**b**,**e**,**h**,**k**): 2 μm; (**c**,**f**,**i**,**l**): 200 nm.

The chemical structure of PBCE, BDG10, and BDG30 polymers was confirmed by 1H-NMR, as previously reported [37]. The as-synthesized polymers were used to produce films by solvent casting and, in the case of the homopolymer, to also prepare compression-moulded films (PBCE*). All the obtained films are opaque and light-yellow colored, most probably due to a minimal amount of residual catalyst employed in the polymer synthesis (Figure 1a^I^,d^I^,g^I^,j^I^).

All films differ in the surface morphology (Figure 1a–l). PBCE shows a microporous structure with many visible voids on the entire area and spherulite-like aggregations (diameter from 10 to 20 μm) (Figure 1d–f). On the other hand, a reduction of the pore size was observed by the introduction of BDG co-units. In particular, the BDG10 surface is characterized by spherulites partially combined resulting in irregular voids (Figure 1g–i), while the BDG30 surface is composed of small aggregates without visible voids, therefore resulting in more compact and smoother than the other two films (Figure 1j–l). PBCE* has markedly different surface morphology than PBCE, its surface being, indeed, free of voids and visible spherulite-like aggregations, and thus resulting the most compact and smooth among all the prepared films (Figure 1a–c).

The prepared films also differ for the surface wettability characteristics. In fact, by copolymerization we achieved an increase of the surface wettability (lower hydrophobicity), as evidenced by the lowering of WCA values with the increment of highly electronegative ether-oxygen atom content (PBCE and BDG30 display WCA = 100° and WCA = 86° respectively, Figure 1f^I^,l^I^, Table 1). In the case of PBCE* film, an opposite effect was achieved: a reduction of the surface wettability, higher hydrophobicity, and higher WCA (110°) (Figure 1c^I^,f^I^,i^I^,l^I^, Table 1). The different wettability of the films was also confirmed by the levels of adsorbed proteins (purified BSA, FBS2%, FBS10%) that increased with the reduction of the WCA in the order PBCE* <<< PBCE < BDG10 < BDG30 (Figure S1a–c).

Thermal analyses revealed that all films are semicrystalline, with a glass transition step and a melting endotherm peak being evident in the calorimetric traces. We have recorded a reduction of both melting enthalpy (ΔH_m_) and temperature (T_m_) with the increase of the BDG content: T_m_ shifts from 167 °C (PBCE) to 120 °C (BDG30), while melting enthalpy decreases from 45 Jg^−1^ for PBCE to 27 Jg^−1^ for BDG30 (Table 1). The glass transition temperature (T_g_), measured after quenching, decreases from 15 °C for PBCE to −17 °C for BDG30. DSC results were confirmed by X-ray diffraction (XRD), as we have observed a decrease in the crystallinity degree (X_c_) with the increase of BDG mol% (Table 1). PBCE* showed a decreased amount of crystallinity with respect to PBCE (42% and 52%, respectively), as expected based on the known effect of solvent-induced crystallization (Table 1).

The overall data indicated that PBCE*, PBCE, BDG10, and BDG30 films differed for surface microstructure and wettability due to the difference in crystal dimension and crystallinity degree. These characteristics make them a suitable platform for the study of the effect of surface property on stem cells.

### 2.2. Culture of hBM-MSCs on PBCE* and PBCE, BDG10, BDG30 Polymer Films

**Figure 2:**
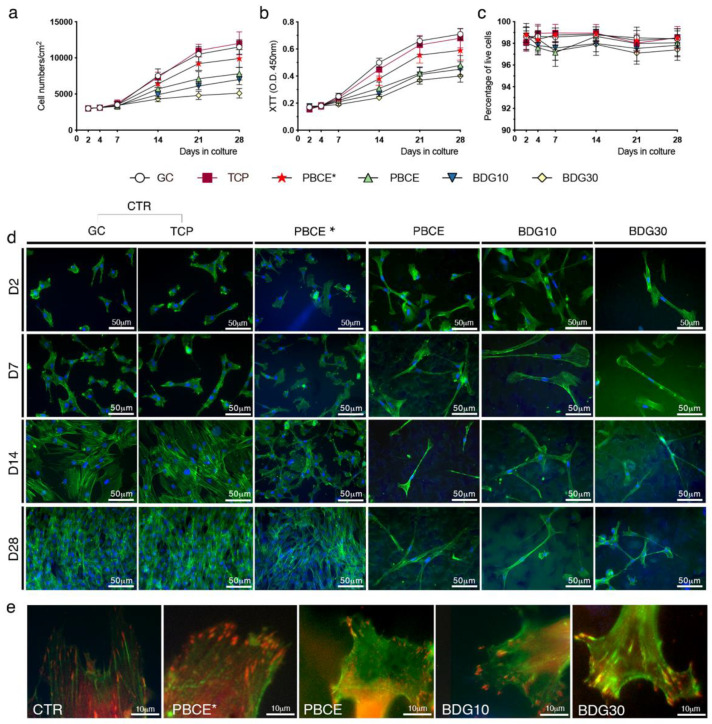
PBCE, BDG10 and BDG30 polymer films, but not PBCE*, change the hBM-MSC shape. (**a**) Growth curves of cell proliferation on polymer films, TCP and GC. (**b**) XTT viability assay showed the absence of cytotoxicity in cells on polymer films, TCP and GC. (**c**) Percentage of live cells on polymer films, TCP and GC at each time point of culture. The results in a,b,c, are expressed as mean ± SD of three independent experiments, *p* < 0.05. (**d**) Representative fluorescence images of hBM-MSC nuclei (DAPI, blue) and F-actin (FITC-phalloidin, green) revealed the stem cell canonical fibroblast-like morphology on TCP and GC and the change of the cell shape only on PBCE-films. Scale bar 50 μm. (**e**) Representative fluorescence images of hBM-MSCs vinculin (red) and F-actin (FITC-phalloidin, green) showed the focal adhesion spots on CTR (TCP/GC) and polymer films. Scale bar 10 μm.

**Figure 3:**
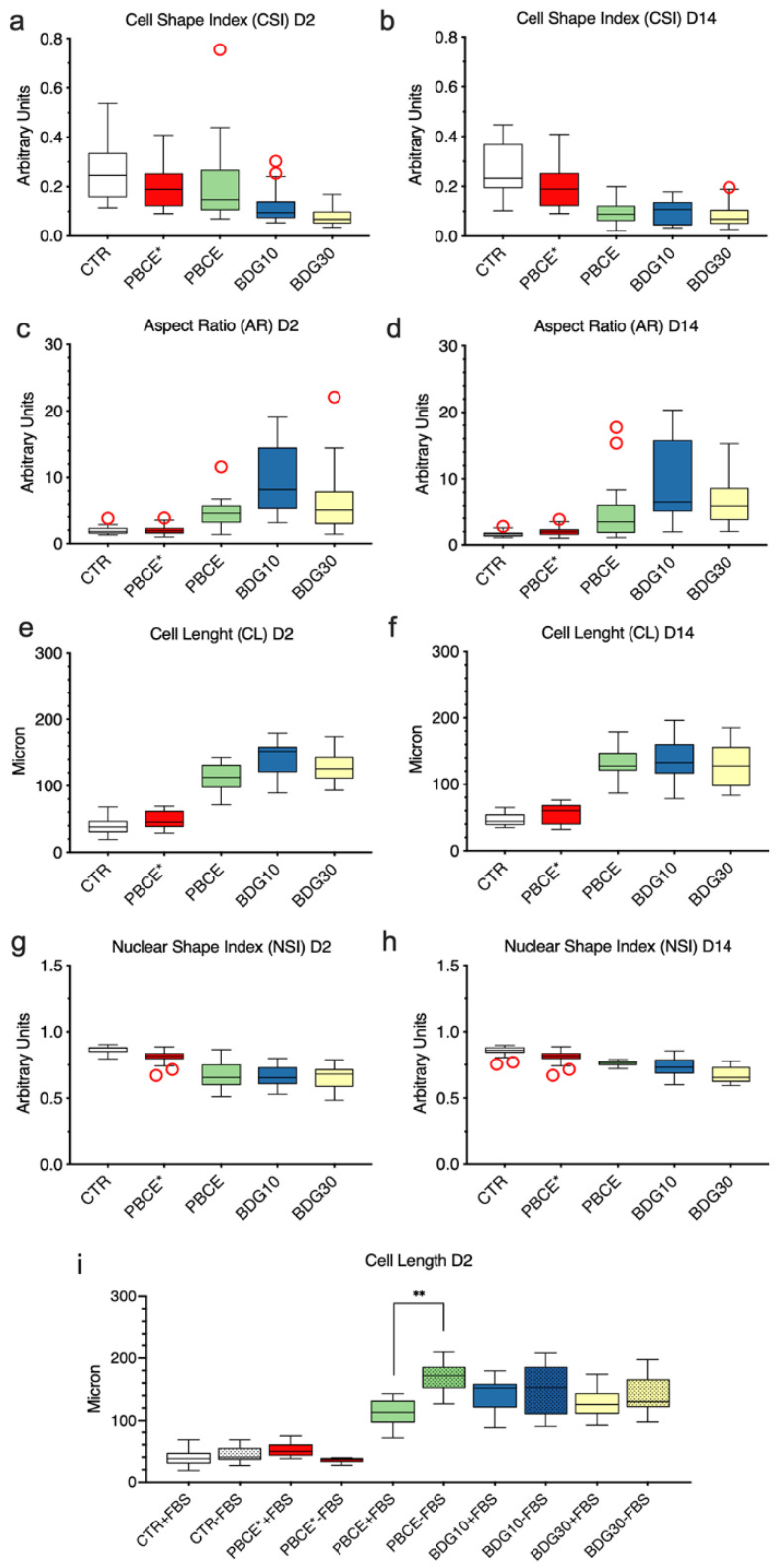
Quantitative morphometric measures of hBM-MSCs on polymer films. Measurements were carried out as described in the methods section at D2 and at D14. (**a**,**b**) cell shape index (CSI); (**c**,**d**) aspect ratio (AR); (**e**,**f**) cell length (CL); (**g**,**h**) nuclear shape index (NSI). (**i**) Comparison of the cell length (CL) between stem cells grown on film- and CTR-cultures on D2 in the presence (+) and absence (−) of FBS. Red circles: outliers. The results are expressed as the mean of three independent experiments, ** *p* < 0.01.

First, we established if PBCE*, PBCE, BDG10, and BDG30 films were suitable for hBM-MSCs culture. We conducted long-term culture studies (28 days [D]) by seeding stem cells on polymer films in a growth culture medium and evaluated the cell proliferation, viability, adhesion, and shape (Figure 2 and Figure 3; Figures S2 and S3). As a reference control, hBM-MSCs were seeded on TCP (canonical culture conditions) and GC (for immunofluorescences and morphometric analysis).

#### 2.2.1. Stem Cells Proliferation and Viability

As previously reported [42] hBM-MSCs adhered to the surface of GC, had a canonical fibroblast-like mesenchymal stem cell morphology and displayed a growth rate and viability comparable to stem cells on TCP (Figure 2a–c,d, CTR [TCP, GC]).

No significant differences were observed in the proliferation rate and cell viability of hBM-MSCs cultured on PBCE* film compared to CTR cultures (Figure 2a–c). In contrast, hBM-MSCs cultured on solvent casted PBCE, BDG10 and BDG30 polymer films (from now PBCE-films) had a lower proliferation rate and reached a plateau phase on D14, whereas hBM-MSCs continued to grow on PBCE* and TCP/GC (Figure 2a). No signs of cytotoxicity were observed in all cultures as demonstrated by the similar curves of the mitochondrial dehydrogenase activity in hBM-MSCs on polymer films and TCP/GC (Figure 2b) and by the comparable viable cells percentage in all systems (Figure 2c).

#### 2.2.2. Stem Cells Shape and Adhesion

We performed a time-course analysis (days: 2, 7, 14, 28) of hBM-MSCs shape on each biomaterial by monitoring the cytoskeleton architecture using F-actin staining (Figure 2d, phalloidin, green). Over time in culture, control stem cells maintained the classical fibroblast-like morphology with the F-actin stress fibers arranged as bundles crossing the cytoplasm in multiple directions along the cell diameter (Figure 2d, CTR) and ending with vinculin spots, thus indicating canonical focal adhesion organization (Figure 2e, CTR).

hBM-MSCs cultured on PBCE* film maintained the fibroblast-like morphology, with nuclei almost round and the F-actin fibers arranged as in CTR cultures (Figure 2d, PBCE*). The presence of Vinculin spots at the ends of F-actin fibers indicated the presence of focal adhesion plaque as in CTR cells (Figure 2e).

hBM-MSCs cultured on PBCE-films lost the fibroblast-like morphology. The phenomenon was rapid on each material, with some differences among films (Figure 2d). On D2 after seeding, stem cells on PBCE had a heterogeneous shape with the majority of cells that retained the mesenchymal morphology while the others (~43% of total cells; Figure S2) acquired a pretty elongated and polarized form consisting of an enlargement of the cell body on one side of the cells and one long narrow protrusion on the opposite side (Figure 2d; Figure S3, high magnification images). The number of polarized elongated cells became more homogeneous at D7 and meanwhile, the length of the cells increased (Figure 2d; Figure S2). This scenario was maintained at D14 and D28 (Figure S2). hBM-MSCs on BDG10 and BDG30 acquired the elongated and polarized shape as on PBCE (Figure 2d; Figure S3, high magnification images), but the phenomenon was faster. In fact, on D2, the percentage of elongated and polarized stem cells was higher on BDG10 (~65% of total cells) and BDG30 (~60% of total cells) with respect to PBCE (Figure S2). This difference disappeared at D7 when almost 70–80% of the cells acquired the new morphology on all PBCE-films (Figure 2d; Figure S2). Changes in cell shape correlated with nuclear positioning since nuclei moved from the basal cytoplasm to the neck of the cell body enlargement (Figure S4). Of note, the morphological changes did not interfere with cell adhesion on the PBCE-films surface as F-actin microfilaments ended with vinculin adhesion spots as in CTR culture (Figure 2e). Finally, polarized and elongated stem cells displayed a random orientation on PBCE-films over time in culture (D2–D28), as shown by the similar Feret angle values at each time point (Figure S5).

#### 2.2.3. Quantitative Morphometric Measurements

To properly correlate the effect of PBCE-films on stem cells, we measured the cell shape index (CSI), nuclear shape index (NSI), cell aspect ratio (AR), and cell length (CL) descriptors. The measurements were performed by using the phalloidin-images of cells on all films. Quantifications were performed at D2 because it is the time point in which we observed differences in terms of heterogeneous/homogeneous population of elongated and polarized cells among films (Figure S2; Figure 3), and at D14 when cells morphology on all PBCE-films became homogeneous (Figure S2; Figure 3). All determinations were carried out also in PBCE*- and CTR-cultures (Figure 3).

The CSI is a measure of the cell spreading area [14,17]. No variations of CSI in PBCE* cultures with respect to the CTR were observed at both time points (Figure 3a,b). In contrast, we quantified a significant CSI reduction in hBM-MSCs cultured on PBCE-films (Figure 3a,b). In fact, with respect to CTR, at D2 the CSI was slightly reduced in stem cells on PBCE (11%), and much more in stem cells on BDG10 (55%) and on BDG30 (69%) (Figure 3a). This gap was balanced at D14 as the CSI reduction was similar in all PBCE-films (PBCE~63%; BDG10~64%; BDG30~68%) (Figure 3b).

The AR descriptor, wich is a measure of the ratio of cell major axis/minor axis, was similar in PBCE* and CTR cultures, whereas it was significantly increased in cells on the other films (Figure 3c,d). Compared to CTR, the increase was in the order BDG10 > BDG30 > PBCE, corresponding to 104% (D2) and 171% (D14) on PBCE, 248% (D2) and 350% (D14) on BDG10, and 144% (D2) and 226% (D14) on BDG30 (Figure 3c,d). We also detected a sharp increase in the length of stem cell major axis (CL) on PBCE-films compared to CTR (Figure 3e,f). The highest elongation of stem cells was on BDG10 (D2: 352%; D14: 355%) compared to BDG30 (D2: 331%; D14: 340%) and PBCE (D2: 297%; D14: 337%) (Figure 3e,f). On D14 stem cells on PBCE-films were slightly longer than at D2 (Figure 3e,f). No CL difference was observed in hBM-MSCs on PBCE* with respect to CTR (Figure 3e,f).

We found a significant reduction of NSI in hBM-MSCs on PBCE-films compared to CTR, both at D2 and at D14, while no variation was observed on PBCE* (Figure 3g,h). The stretching of the nuclei might be the consequence of cell polarization and elongation together with the spatial nuclear positioning inside the cells (Figure 3g,h; Figure S4a). We observed a comparable nuclear displacement along the cell protrusion direction in all polymer-cultures at both D2 and D14 on PBCE-films (Figure S4a). Collectively, data presented in this section demonstrated that (i) PBCE-films caused a drastic change of the shape of hBM-MSCs; (ii) the inclusion of BDG-units into PBCE accelerated the phenomenon; (iii) hBM-MSCs cultured on PBCE* maintained mesenchymal morphology and behavior. We suggest that these effects are the consequence of the different films’ surface microstructure and wettability, which in turn are dependent on the film preparation.

### 2.3. Elongation and Polarization of hBM-MSCs on PBCE-Films Are the Consequence of Coordinate Action of F-Actin Fibers and Microtubules

To investigate how the hBM-MSCs morphological changes on PBCE-films occur, we cultured stem cells on PBCE*, PBCE, BDG10, and BDG30 in a growth culture medium in the absence of FBS. These culture conditions excluded the potential influence of FBS proteins adsorbed to films surface on the stem cell fate [21,23]. The analyses were conducted at D2 because it was the shortest time point at which differences were noticed among polymers cultures. Experiments in the presence of FBS were also conducted. For comparison, the analyses were also performed on PBCE* and on CTR systems in the presence/absence of FBS.

#### 2.3.1. Culture of hBM-MSCs on Polymer Films in the Absence of FBS

**Figure 4:**
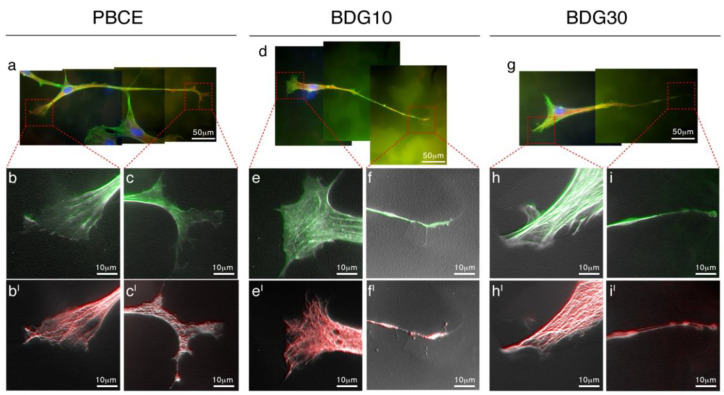
Coordinate nucleation of F-actin and Microtubules drives the hBM-MSCs’ shape on PBCE, BDG10 and BDG30. (**a**,**d**,**g**) Representative fluorescence images of hBM-MSC in proliferation medium in the absence of FBS (F-actin [FITC-phalloidin, green], Microtubules [human anti-tubulin antibody, red], and nuclei [DAPI, blue]). Due to the length of the cells, the images include 2/3 microscope frames. Elaboration of selected details ((**b**,**c**,**b^I^**,**c^I^**); (**e**,**f**,**e^I^**,**f^I^**); (**h**,**i,h^I^**,**i^I^**)) revealed the coordinate action of F-actin and tubulin in building the new cytoskeleton architecture of the cell as a consequence of the seeding on polymer films.

No stem cell elongation (Figure 3i), nor polarization or other morphological changes were detected on PBCE* or in CTR systems in the absence of FBS (Figure S6). Indeed, only a very small number of cells were found to adhere to the PBCE* surface in these culture conditions, and already at D2 cells were almost detached from the film surface (Figure S6). On the contrary, even in the absence of FBS stem cells are elongated and polarized (cell body enlargement on one pole of the cells and emission of the cell protrusion on the opposite side) on PBCE-films (Figure 3i and Figure 4a,d,g, Figure S6).

The CL was higher in cells on PBCE rather than on the other PBCE-films (order: PBCE > BDG10 > BDG30), contrasting the same measures obtained in parallel experiments in the presence of FBS (order: PBCE < BDG30 < BDG10) (Figure 3i). The displacement of nuclei was similar in stem cells on PBCE-films cultured without FBS and was also comparable with that measured in the presence of FBS at D2 (Figure S4a,b).

#### 2.3.2. Coordinated Action of F-Actin Fibers and Microtubules Generates the New Cell Shape

Next, we examined the architecture of the cytoskeleton fibers in cells on PBCE-films, cultured without FBS through the images’ computational analysis of F-actin filaments and microtubules (MTs) molecular organization (Figure 4). The whole-cell morphology was similar among PBCE-films with some differences in the cell edges (Figure 4). The latter are the regions where the cytoskeleton fibers grow and branch out, therefore, we focused the attention on the description of the terming ends. Details of the edges conformation in cells on PBCE showed the F-actin fibers arranged in parallel orientation in the cell body enlargement and set out in veil/bundles lamellipodia with branched spinet filopodia in the cell protrusion (Figure 4b,c). Both structures served as a guide for MTs bundles, that retraced the F-actin microfilament organization (Figure 4b^I^,c^I^). Edges magnification in BDG10-cell cultures revealed F-actin fibers organized parallelly and ventrally with several networks in the cell body enlargement culminating with lamellipodia, and showed parallel oriented microfilaments that ended with a thin branched filopodium in the cell protrusion (Figure 4e,f). Again, the microfilaments acted as a guide for the MTs bundles that mirrored the F-actin architecture (Figure 4e^I^,f^I^). Finally, the edges in BDG30-cell cultures had F-actin fibers parallelly and ventrally orientated in the cell body enlargement that ended with short branched filopodia and a veil/bundles lamellipodium in the cell protrusion (Figure 4h,i). Yet the microfilaments served as a guide for MTs bundles but, in several cases, we observed small branches that did not overlap the F-actin in the terminal side of cells protrusion (Figure 4h^I^,i^I^). Of note, the results in Figure 4 are also representative of the cytoskeleton architecture in cells on polymer films in the presence of FBS on D2.

#### 2.3.3. Interaction of hBM-MSCs with PBCE, BDG10 and BDG30 Polymer Films

**Figure 5:**
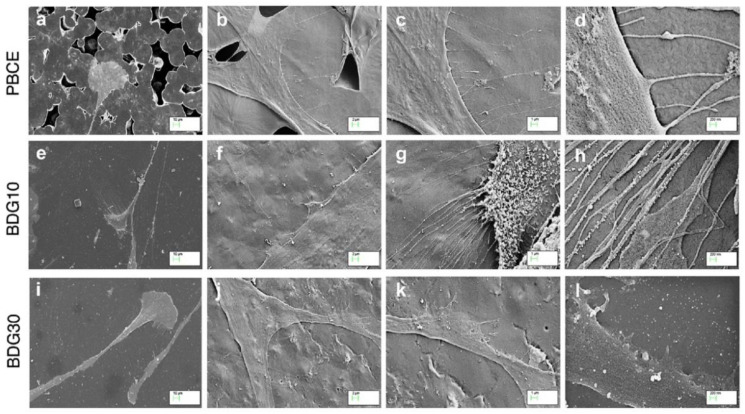
Interaction of hBM-MSCs with the surface of PBCE, BDG10 and BDG30. FESEM representative images at different magnifications ((**a**,**e**,**i**): 10 μm; (**b**,**f**,**j**): 2 μm; (**c**,**g**,**k**): 1 μm; (**d**,**h**,**l**): 200 nm) revealed the interaction of hBM-MSCs on polymer films surfaces.

Representative FESEM images confirmed the elongated and polarized cell shape on solvent casted films (Figure 5a,e,i) and highlighted the different interactions of stem cells with the surface microstructure of each film (Figure 5b–d,f–h,j–l). Specifically, we observed that hBM-MSCs interacted with: (i) PBCE surface by extending thin, spaced and slight filopodia (Figure 5b–d); (ii) BDG10 surface by developing clumps of short filopodia (Figure 5f–h); (iii) BDG30 surface by spreading short and broad lamellipodia (Figure 5j–l).

Overall, the results suggest that the new cell morphology is the consequence of the simultaneous and coordinated response of the F-actin microfilaments and MTs to the PBCE-films, and point out that the surface microstructure and wettability of polymer matrix are critical in inducing the stem cell shape change, as shown by the higher stem cell elongation and cell protrusion length on PBCE compared to BDG10 and BDG30 in the absence of FBS.

### 2.4. Is the Long Protrusion a Sign of Neuronal-Like Progenitor Cell Transitions of hBM-MSCs on Polymer Films?

**Figure 6:**
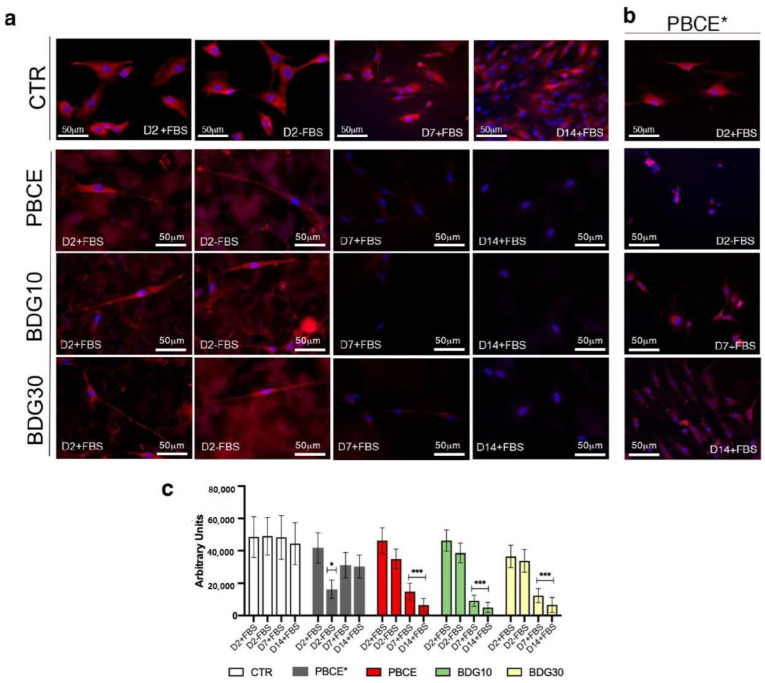
Expression of nestin in hBM-MSCs grown on PBCE*, PBCE, BDG10 and BDG30. Representative fluorescence images revealed the expression of nestin in stem cells in (**a**) CTR cells, PBCE-, BDG10- and BDG30-polymer cultures at D2 (in the presence (+) and absence (−) of FBS), at D7 and D14 (in the presence (+) of FBS); (**b**) cells on PBCE* at D2 (in the presence (+) and absence (−) of FBS), at D7 and D14 (in the presence (+) of FBS). Nuclei (DAPI, blue), and nestin (human anti-nestin antibody, red). Scale bar 50 μm. **(c)** quantitative analysis of nestin expression in hBM-MSCs on PBCE*, PBCE, BDG10 and BDG30 a CTR at each time point. Data are expressed as mean ± SD, * *p* < 0.05, *** *p* < 0.005. For each experiment, an average of 100 cells for each culture condition was considered.

**Figure 7:**
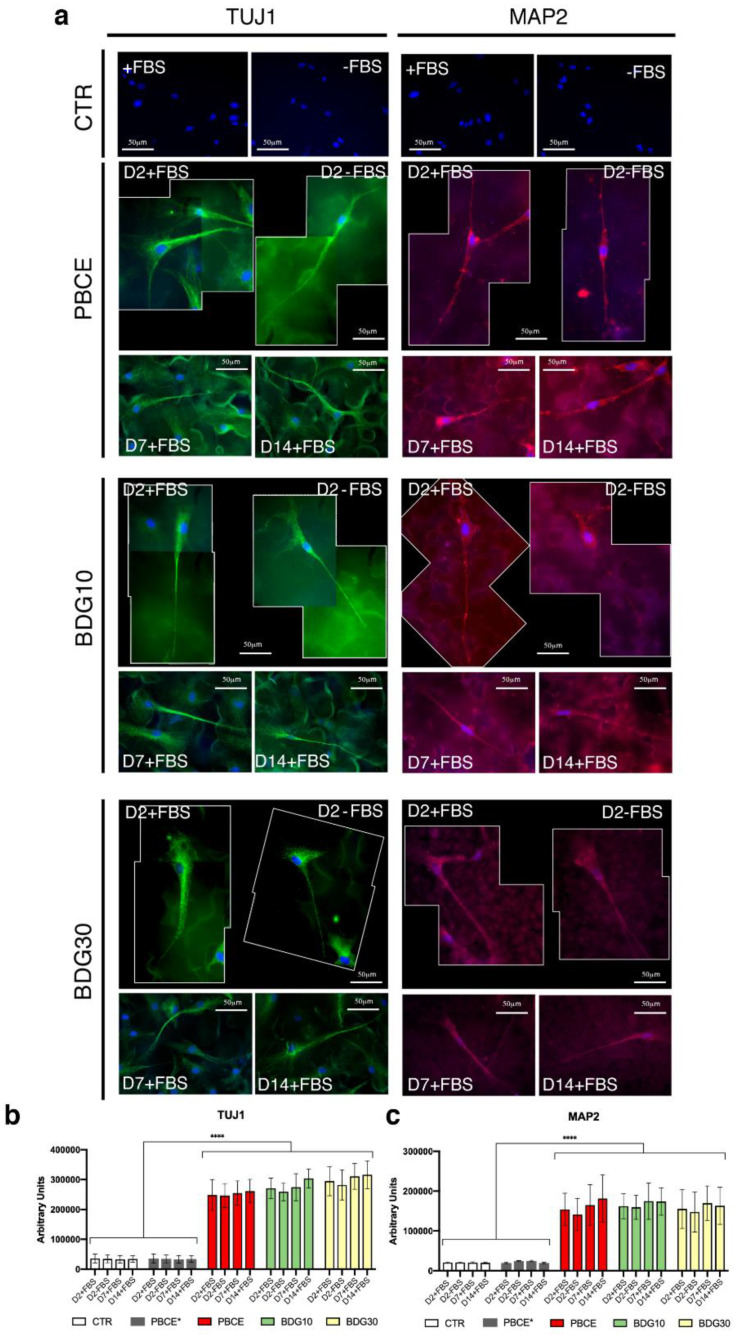
Expression of TUJ1 and MAP2 in hBM-MSCs grown on PBCE, BDG10 and BDG30. (**a**) Representative fluorescence images of hBM-MSC nuclei (DAPI, blue), TUJ1 (human anti-TJU1 antibody, green), and MAP2 (human anti-MAP2 antibody, red), revealed the absence of TUJ1 and MAP2 in cells on CTR cultures, both in FBS presence (+) and absence (−). TUJ1 expression in stem cells on PBCE, on BDG10 and on BDG30 at different time points (D2-D14 in the presence (+) FBS and at D2 and in the absence (−) of FBS). MAP2 expression in stem cells on PBCE, on BDG10 and on BDG30 at different time points [D2-D14 in the presence (+) FBS and at D2 in the absence (−) of FBS]. Scale bar 50 μm. Due to the length of the cells, the images include two microscope frames. The CTR images are representative of all time points analyzed (D2-D14). See also Figure S7 in supporting file for visual images in gray levels. Fluorescent interference of polymer films without cells was evaluated. (**b**,**c**) Quantitative IF of the expression of TUJ1 (**b**) and MAP2 (**c**) in hBM-MSCs grown on PBCE, BDG10, BDG30, PBCE* and in control condition at each time in culture. Data are expressed as mean ± SD, **** *p* < 0.0001. For each experiment, an average of 100 cells for each culture condition was considered.

**Figure 8:**
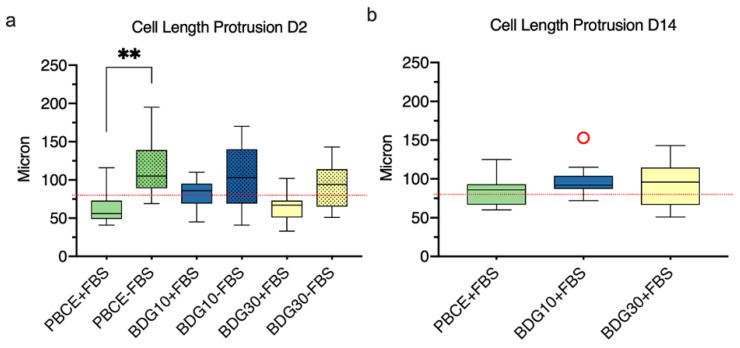
Quantitative measurements of the cell protrusion on PBCE, BDG10 and BDG30. (**a**) Comparison of the cell protrusion length between stem cells grown on PBCE-films and CTR cultures on D2 in proliferation medium in the presence (+) and absence (−) of FBS. (**b**) Measurement of cell protrusion length in stem cells grown on polymer films on D14 in proliferation medium in the presence of FBS. Red circles: outliers. The results are expressed as the mean of three independent experiments, ** *p* < 0.01.

Finally, we explored if the change of stem cell shape on polymer films matched with a peculiar stem cell lineage commitment and differentiation. We have documented that stem cells polarized and extended one long cell protrusion on all PBCE-films (Figure 2, Figure 3, Figure 4 and Figure 5). This cell morphology mimics the neuronal cell shape. Therefore, we analyzed the expression of several neuronal markers associated with the stages of (i) very early neural progenitor stem cells (nestin), (ii) late neuronal precursor (neuron-specific class III β-tubulin [TUJ1]) and (iii) mature neuron (microtubule-associated protein 2 [MAP2] and [TUJ1]) [53]. We also analyzed the expression of the glial fibrillary acidic protein (GFAP; astrocytes) and chondroitin sulfate proteoglycan 4 (NG2; oligodendrocytes) glial markers [53]. Experiments were conducted by seeding hBM-MSCs on PBCE, BDG10, BDG30 in proliferation medium in the presence/absence of FBS on D2 and in the presence of FBS on D7 and D14 (Figure 6, Figure 7 and Figure 8; Figures S7 and S8). Comparative experiments were performed in stem cells seeded on PBCE* and CTR (Figure 6 and Figure 7; Figures S8 and S9).

#### 2.4.1. Nestin Is Downregulated in hBM-MSCs Seeded on PBCE, BDG10 and BDG30

hBM-MSCs on CTR system expressed the nestin marker in the presence/absence of FBS and for the whole culturing time at comparable levels as showed in the representative immunofluorescence images (Figure 6a,b) and by quantitative analyses (Figure 6c). These results agreed with previously published reports that considered the expression of nestin a marker of undifferentiated mesenchymal stem cells [54,55,56,57]. We found a time-dependent expression of nestin in cells on PBCE-films as showed in Figure 6a (representative immunofluorescence images) and by quantitative analyses in Figure 6c. We found a comparable expression of nestin in cells on PBCE-films with respect to stem cells on CTR at D2, both in the presence/absence of FBS (Figure 6a,c, D2+FBS/D2-FBS), while the expression of the marker was strongly reduced at D7 and became almost undetectable at D14 (Figure 6a,c, D7+FBS/D14+FBS). Conversely, hBM-MSCs on PBCE* expressed nestin at each time point analyzed, although the level was slightly decreased with respect to control cells at D7, and then was maintained constant at D14 (Figure 6b,c). The low level of nestin expression at D2-FBS was due to the adverse effect of PBCE* on stem cell viability in this culture condition (Figure S6).

#### 2.4.2. hBM-MSCs Seeded on PBCE, BDG10, BDG30 Express TUJ1 and MAP2

Cells expressed the neuronal marker TUJ1 on PBCE-films in the presence of FBS at D2. The TUJ1 staining recapitulated the elongate cell shape, highlighting both the cell body enlargement and the cell protrusion (Figure 7a,b; TUJ1 D2 + FBS; Figure S7). The TUJ1 expression persisted on D7 and D14 in culture with FBS on PBCE-films (Figure 7a,b; TUJ1, D7, D14; Figure S7). Of note, TUJ1 was also expressed in all PBCE-films at D2 in the absence of FBS (Figure 7a,b; TUJ1; D2-FBS), while no expression was observed in cells on PBCE* (Figure 7b, Figure S9) and CTR (Figure 7a,b). The quantification of the TUJ1 expression confirmed the high expression of this marker at D2 with its stabilization at D7 and D14 (Figure 7b), and highlighted the high expression of TUJ1 in cells on all PBCE-films at D2 also in the absence of FBS (Figure 7b).

Cells also expressed the neuronal marker MAP2 in the presence of FBS at D2 and the expression persisted over time in cultures on PBCE-films (Figure 7a,c; D2 + FBS; D7, D14; Figure S7). The signal was also observed in the absence of FBS on D2 in all PBCE-films (Figure 7a,c; D2-FBS). Yet, no MAP2 expression was observed in cells on PBCE* (Figure 7c, Figure S9) and CTR (Figure 7a,c).

As above, the quantification of the MAP2 expression confirmed its high expression at D2 and its steady expression on PBCE-films at D7 and D14 (Figure 7c). The analysis also indicated the high expression of MAP2 in cells on all PBCE-films at D2 in the absence of FBS (Figure 7c).

Finally, we monitored the expression of the glial markers (Figure S8). No expression of GFAP and NG2 was detected in all PBCE-films cultures at D2 in the presence/absence of FBS, as well as at D7 and D14 in the growth culture medium (Figure S8).

Of note, no GFAP^+^ or NG2^+^ cells were detected in PBCE*-cultures (Figure S9) nor in CTR over the culture time and in both experimental conditions (Figure S8).

#### 2.4.3. Stem Cells Protrusion Measurements

The measurements of the cell protrusion length supported the neuronal-like differentiation of hBM-MSCs on PBCE-films (Figure 8). The analysis was performed at D2 in the presence/absence of FBS and on D14 in the presence of FBS.

The length of the cell protrusion followed the CL order (Figure 8; Figure 3e,f,i). Thus, on D2 in the presence of FBS, the protrusion length ranged from 41 μm to 116 μm on PBCE, from 45 μm to 110 μm on BDG10 and from 33 μm to 102 μm on BDG30, whereas in the absence of FBS, it ranged from 69 μm to 195 μm on PBCE, from 41 μm to 170 μm on BDG10, and from 51 μm to 143 μm on BDG30 (Figure 8a). These data indicated that the length of cell protrusion at D2, in polymer cultures without FBS was always higher than those measured in the presence of FBS (Figure 8a). Finally, on D14, the cell protrusion length was higher than that at D2 in the presence of FBS and the length was almost above 80 μm on all substrates, reaching 100–150 μm (Figure 8b).

Overall, results suggested that hBM-MSCs on PBCE-films were induced toward a neuronal-like phenotype, while the differentiation process was absent on PBCE*.

## 3. Discussion

In this work, we have demonstrated that hBM-MSCs cultured on PBCE-films in the growth culture medium is induced toward a neuronal-like phenotype. The phenomenon was frightfully fast, since at D2 of culture on PBCE-films hBM-MSCs lost the canonical mesenchymal morphology, lengthen significantly, and acquired a polarized shape with an enlargement of the cell body on one side and the extension of one long cell protrusion on the opposite side. This morphology mirrored the canonical neuron shape and was supported by the expression of TUJ1 and MAP2 neuronal markers. Of note, on PBCE* film the phenomenon was not observed as hBM-MSCs maintained the stem cell mesenchymal shape and behavior and did not express neuronal or other neural markers.

These findings were specific for hBM-MSCs as human fibroblasts maintained their canonical features in long-time culture (27 days) on neat PBCE, BDG10 and BDG30 polymer films, as reported in our previous work [58].

We interpreted these results as the consequence of the cross-talk between hBM-MSCs and the surface microstructure and wettability of the above-mentioned films.

PBCE and PBCE* are the same polymeric matrix which, however, based on the processing technique adopted (solvent casting and compression-moulding, respectively), produce films with different surface microstructure characteristics and wettability. It’s well known that the solvent casting procedure facilitates the polymer crystallization process with a consequent increase in the amount of crystalline phase, which is responsible for the roughness of the film surface. That explains why PBCE film is characterized by a rough microstructure surface while, on the contrary, PBCE* film has a smooth one. Moreover, it has to be taken into account that both PBCE and PBCE-based copolymers crystallize according to a spherulitic morphology, as usually happens in polyesters [37,45]. Spherulite dimensions are related to the crystal perfection degree, which can be finely tuned, together with the amount of developed crystalline phase, by copolymerization. Specifically, the increase in the amount of co-units introduced along the macromolecular chain corresponds to a lower and less perfect crystalline phase, which results in a smoother surface. Therefore, only the PBCE surface characteristics, roughness/voids microstructures and increased wettability (WCA = 100°) rather than smoothness/compact microstructure and low wettability (WCA = 110°) of PBCE* were able to trigger the induction of the neuronal-like commitment, indicating that the stem cells discriminate between these different surface characteristics and respond accordingly by activating the neuronal differentiation program. These findings were confirmed and enhanced by the stem cell neuronal-like differentiation observed in BDG10 and BDG30 films. It’s worth noting that by copolymerization, we were able to act simultaneously on surface morphology and wettability, this latter tunable by changing the amount of ether-oxygen atoms along the polymer chains (i.e., the higher the BDG co-units content, the more hydrophilic the film surface). Specifically, the introduction of ether linkages along the PBCE backbone led to (i) a reduction of the degree of crystallinity and, in turn, the surface irregularities, according to the trend BDG30 < BDG10 < PBCE and (ii) a concomitant and progressive increase of surface wettability, i.e., BDG30 > BDG10 > PBCE. The best performance in terms of the highest number of elongated and polarized cells and expression of neuronal markers on D2, was observed on BDG10 (order: BDG10 > BDG30 > PBCE) suggesting that in this film the best combination of microstructure characteristics and wettability degree was obtained. Interestingly, in parallel experiments, the effectiveness of PBCE-films surface microstructure and wettability on inducing neuronal-like commitment at D2 was highlighted in FBS-free medium culture conditions. In this case, the best performance was observed on PBCE film, further confirming that neat PBCE film had the properties for steering the stem cell toward the neuronal fate.

Thus, the first sign of the neuronal-like commitment was observed at D2 of stem cells culture on PBCE-films and consisted in the acquisition of a new shape with cells highly elongated (~3-4 fold-increase with respect to CTR and PBCE*) and polarized with a cell body enlargement on one side and with an extension of one cell protrusion on the opposite side. The new cell shape is obtained by the new architecture of the cytoskeleton fibers, both F-actin and microtubules, that are induced to increase the polymerization and remodeling activities in a coordinate manner as a consequence of the cellular response to the films surface characteristics. This is a clear example of the cell tensegrity mechanism [10,11,14], as stem cells adapted their cytoskeleton to the new surface where the cells were seeded [10,11,14]. The correlation between cell shape, cellular phenotype, and behavior have been intensively investigated [59]. However, only in the past decade, the biomolecular correlation has been demonstrated [17,29,59,60,61]. Thus, it is now accepted that the cell shape may be informative of the cell functions and phenotype likewise molecular signaling [14,15,60]. Therefore, the control of cell shape through material engineering represents an efficient alternative strategy to modulate cell functions. In PBCE-films, the cytoskeleton architecture depicted the cell neuronal-like morphology and this agreed with the expression of the neural lineage markers [53,62,63,64,65] nestin (early neural progenitor), TUJ1 (neuronal precursor and mature neuron), MAP2 (mature neuron), and the absence of expression of GFAP (astrocytes) and NG2 (oligodendrocytes) markers. Of note, the expression of nestin has been documented in hBM-MSCs and is considered as a marker of undifferentiated stem cells [54,55,57]. We found comparable expression markers in hBM-MSCs on PBCE-films, as cells were nestin^+^TUJ1^+^MAP2^+^GFAP^-^NG2^-^ at D2 and nestin^low^TUJ1^+^MAP2^+^GFAP^-^NG2^-^ at D7 and D14. On the contrary, stem cells were nestin^+^TUJ1^-^MAP2^-^GFAP^-^NG2^-^ at D2/D7/D14 on PBCE*, and therefore closer to the stem cell behavior observed in the control system. Indeed, the level of nestin was slightly lower in stem cells on PBCE* compared to the control. We interpreted this result as a confirmation of the effect of the PBCE molecule on stem cells, and as well as of the ineffectiveness of PBCE* to trigger the differentiation program as a consequence of the diverse film characteristics with respect to the other PBCE-films. The simultaneous expression of TUJ1 and MAP2 and the absence of expression of the GFAP and NG2 glial markers indicated that hBM-MSCs were steered toward a neuronal-like phenotype on PBCE-films. The phenotype transition was observed at D2, but the concomitant expression of the early neuronal progenitor marker, together with that of precursor and mature neuron markers indicated that, at this stage, the neuronal commitment had just begun. Likewise, the reduction of the expression of nestin and the sustained expression of TUJ1 and MAP2 at D7 showed that the neural commitment is driven to the neuronal-like phenotype. Noteworthy, even in FBS-free growth medium, stem cells were nestin^+^TUJ1^+^MAP2^+^GFAP^-^NG2^-^ at D2 on all PBCE-films, further confirming that the microstructure and wettability of the materials trigger the neuronal-like commitment.

Many reports have demonstrated the differentiation of hBM-MSCs towards neural lineage either in in-vitro culture [66,67,68] or on biomaterials [42,69,70,71]. For instance, hBM-MSCs seeded on the hydrophobic diphenylamino-s-triazine-bridged p-phenylene-coated surface were steered toward neurosphere-like cellular aggregates and then induced to differentiate into neural cells in the presence of neural medium [69]. More recently, a three-dimensional nanostructured microarchitecture efficiently induced hBM-MSCs to align and to acquire neurogenic differentiation (over 85% of hMSCs express MAP2), after 7 days of culture in the growth culture medium [70]. Interestingly, in the published works, the MSC neuronal differentiation has been obtained in films/scaffold with a patterned surface, mainly with channel topographies [42,69,70,72,73], therefore in this regard, our results are new as they demonstrated the neuronal differentiation in unpatterned surface polymer films.

Furthermore, to the best of our knowledge, the neuronal-like commitment of hBM-MSCs after only 2 days of culture in the growth medium, even in the absence of FBS, has never been observed so far. The high neuronal induction efficiency of PBCE-films is confirmed by the expression of MAP2^+^ and TUJ1^+^ markers and by the concomitant immediate down-regulation of nestin markers. We suggest that stem cells feel the unpatterned microstructure and wettability characteristics of the polymer surface and respond with a reorganization of the cytoskeletal components, which result in cell reprogramming toward the neuronal lineage. However, further experiments are needed to show the functional activity of generated neuronal-like cells.

Finally, the high efficiency of this platform suggests that it might be useful for establishing cell models for the evaluation of treatment for neurodegenerative diseases with hBM-MSCs as candidates based on the multipotency characteristic of these cells on generating non-hemopoietic stem cells (including neural cells) also in vivo transplantation application.

## 4. Materials and Methods

### 4.1. PBCE-Based Polymer Synthesis

Poly(butylene 1,4-cyclohexanedicarboxylate) and two poly(butylene 1,4-cyclohexane dicarboxylate/diglycolate) random copolymers, containing 10 mol% (BDG10) and 30 mol% (BDG30) of butylene diglycolate co-units, respectively, were synthesized by bulk two step melt polycondensation, as previously reported [37]. These molar compositions have been chosen to reduce the surface hydrophobicity of the film thanks to the presence of ether-oxygen atom containing co-units (BDG) yet maintaining a sufficiently high crystal fraction and perfection to guarantee mechanical stability of polymer film.

### 4.2. PBCE-Based Polymer Preparation

Solvent Casting processing. Free standing films of poly(butylene 1,4-cyclohexanedicarboxylate) (PBCE), BDG10 and BDG30 (about 90 μm thick) have been obtained by solvent casting in chloroform (CHCl_3_) (polymer/solvent ratio was chosen as 5% wt/v). After complete polymer dissolution, the mixture was cast onto a glass Petri substrate and air dried at room temperature (RT) for 24 h and additional 48 h in vacuum.

Compression Moulding processing. Poly(butylene 1,4-cyclohexanedicarboxylate) homopolymer films (200 μm thick) were also developed by compression moulding (namely PBCE*). The polymeric powders were placed between thin Teflon plates (0.3 mm thick), with an appropriate spacer, and heated at T = Tm + 40 °C for 2 min under a pressure of 2 ton/m^2^. Finally, hot-pressed films were cooled down to room temperature [37].

### 4.3. PBCE-Based Polymer Films Characterization

All films were characterized in terms of surface, morphological and thermal features. The surface microstructure was evaluated by field emission scanning electron microscopy (FESEM, Supra 25 Zeiss, Baden-Württemberg, Germany). Samples were gold coated with an Agar automatic sputter coater and then analyzed.

Water contact angle (WCA) was assessed by the sessile drop method in air using a KSV CAM101 instrument (KSV Instruments Ltd., Helsinki, Finland).

To determine the thermal transitions a DSC7 calorimeter (Perkin Elmer, Waltham, MA, USA) was used under the following conditions: heating at 20 °C min^−1^ from −120 °C to 40 °C above fusion temperature (I scan), holding for 3 min, quenching to −80 °C (100 °C min^-1^) and heating to 40 °C above the melting point at 20 °C min^−1^ (II scan). X-ray diffraction (XRD) patterns of polymeric films were carried out by using an X’PertPro diffractometer (PANalytical, Malvern, UK) equipped with a fast-solid state X’Celerator detector and a copper target (λ = 0.15418 nm). Data were acquired at each 0.10° step for 100 s in the 5–60° 2θ interval.

### 4.4. Protein Adsorption Assay

Protein adsorption assessments were performed according to our protocol [42] by transferring 2 mg/mL of bovine serum albumin (BSA) (Sigma-Aldrich, St. Louis, MO, USA), 2% fetal bovine serum (FBS, Euroclone S.p.A, Pero (MI), Italy) and 10% FBS on each polymer film (1 cm^2^ square) and control experiments were carried out by incubating 1 cm^2^ square of PBCE, BDG10 and BDG30 with H_2_0d. Proteins were incubated for 30 min and 24 h at 37 °C. After three washing steps in sterile water, total protein content was measured by the Bradford method [74]. Absorbance (595 nm) was measured using a microtiter plate reader (ELISA reader, DV990BV6, GDV, Roma, Italy). Each sample was analyzed in three independent experiments and run in triplicate.

### 4.5. Human Adult Bone Marrow-Mesenchymal Stem Cells Isolation and In Vitro Culture

hBM-MSCs were isolated and cultured as previously described [17,21,42,43]. Briefly, hBM-MSCs were isolated from the waste samples from surgery consisting of bone marrow extracted throughout washouts of the femurs’ medullary cavities of adult donor subjects undergoing primary total hip replacement. The procedure of isolation of stem cells is occasional and is not part of a specific project. All procedures were done after the consent of donors (60-year-old) and were in accordance with the Declaration of Helsinki.

Donor subjects gave informed consent and the institutional ethical committee approved the procedures. After density gradient on Lympholyte (Cedarlane Laboratories Limited, Hornby, ON, Canada), mononuclear cells were isolated from the bone marrow and were seeded in culture flasks in growth medium consisting of RPMI-1640 (Euroclone, Milano, Italy) medium containing FBS 10%, 2 mM L-glutamine, and 1% penicillin–streptomycin (Euroclone) in a humidified atmosphere and 5% CO_2_ at 37 °C. After 5 to 7 days, the non-adherent cells were removed, and fresh medium was added to the flasks. After 15 days, a fibroblast-like colony started to grow. The medium was changed every three days [17,21,42,43]. The mesenchymal phenotype of hBM-MSCs was analyzed by flow cytometry FACScan (BD Biosciences, San Jose, CA, USA ) as previously described [21]. The human anti-CD44, -CD45, -CD73, -CD90, -CD105, human leukocyte antigen (HLA)-ABC (BD Biosciences) were used. Data analyses were performed with the FlowJo software (Tree Star, Ashland, OR, USA). Cells were electronically gated according to light-scattering properties to discriminate cell debris. Isotype-matched nonspecific antibodies were used as the negative control.

### 4.6. Culture of Human Adult Bone Marrow-Mesenchymal Stem Cells on PBCE*, PBCE, BDG10, BDG30 Films

PBCE*, PBCE, BDG10 and BDG30 films were cut in 1 cm^2^ squares, sterilized by three immersions in 70% ethanol for 30 s and 5 rinses in PBS and then were deposited in a 24-well plate. The stem cell suspension was seeded dropwise on sterilized films. After 45 min the growth culture medium was gently added to each film snippet. Stem cell-PBCE* and -PBCE and -BDG10 and –BDG30 cultures were incubated under the canonical condition at 37 °C in a humidified atmosphere with 5% CO_2_. The medium was changed every three days. As the internal control, experiments were performed seeding the same number of stem cells on tissue culture polystyrene (TCP) and glass coverslip (GC). Cultures were performed at different time points (from day 0 to day 28) and evaluated for viability, morphology, cyto-morphometry, adhesion, cell-film interaction and differentiation. Some experiments were performed seeding stem cells on polymer films in the culture medium without FBS and analyzed on day 2.

### 4.7. Stem Cells Growth and Viability Assay

Stem cell growth and viability [17,21,42,43] were evaluated seeding 3 × 10^3^ cells on PBCE*, PBCE, BGD10 and BDG30 (1 cm^2^ square) at different time points 2, 4, 7, 14, 21, 28 days. As the internal control, experiments were performed seeding the same number of stem cells on TCP and GC.

Stem cell growth was evaluated with the Trypan Blue Solution, 0.4% (Invitrogen™, Grand Island, NY, USA ) according to the manufacture’s recommendation for adherent cells. At each time point, stem cells were counted with the hemocytometer by using the Countess™ Automated Cell Counter.

Stem cell viability was performed incubating cell-film cultures with XTT (2,3-bis [2-methoxy-4-nitro-5-sulfophenyl]-2*H*-tetrazolium-5-carboxyanilide inner salt) (Sigma-Aldrich) according to the manufacturer’s recommendation. As the internal control, experiments were conducted seeding stem cells on TCP and GC. Interference effects of PBCE, BGD10 and BDG30 squares without cells on XTT assay were also considered. The absorbance of the samples was measured using a microtiter plate reader (ELISA reader, DV990BV6, GDV, Roma, Italy) at 450 nm with a reference wavelength of 650 nm.

### 4.8. Immunofluorescences

Immunostaining was performed as previously described [17,21,42,43,75]. Briefly, stem cells on PBCE*, PBCE, BGD10 and BDG30 squares, and on TCP and GC were rinsed twice with PBS, fixed in 4% paraformaldehyde for 20 min, washed twice with PBS, and permeabilized and blocked (PBS + 3% FBS, 0.3% Triton X-100) for 1 h at RT. Samples were incubated with phalloidin (Alexa-fluor-488 phalloidin, Invitrogen) for 20 min at RT, or overnight at 4 °C with the primary human antibodies: anti-MAP2 (Abnova, Taipei, Taiwan), anti-GFAP, anti-NG2 (Millipore, Billerica, MA, USA), anti-TJU1, anti-nestin, and anti-βTubulin (Santa Cruz Biotechnology, Santa Cruz, CA, USA). In the latter cases, after 3 washing with PBS, the staining with Alexa-Fluor 488-nm or Alexa-Fluor 594-nm conjugated secondary antibodies (Invitrogen) for 1 h at room temperature was performed. After being washed with PBS, samples were mounted and nuclei were counterstained with Vectashield with DAPI (Vector Laboratories Inc., Burlingame, CA, USA). Images were acquired using fluorescence microscopy (Eclipse-TE2000-S, Nikon, Tokyo, Japan) equipped with the F-ViewII FireWire camera (Soft Imaging System, Olympus, Münster, Germany). Images were elaborated as described below. Interference of PBCE*, PBCE, BDG10 and BDG30 squares without cells to a fluorescence microscope (Eclipse-TE2000-S) was evaluated.

### 4.9. Image Analysis and Computational Quantitative Cyto-Morphometric Measures

Fluorescent stained images were used to calculate the parameters and morphometric descriptors by Fiji software (Fiji Life-Line version, 30 May 2017), as previously described [14]. Images were acquired using a fluorescence microscope (Eclipse-TE2000-S, Nikon) equipped with the F-ViewII FireWire camera (Soft Imaging System, Olympus).

For the morphometric descriptors analysis, eight different areas were photographed (20X magnification, x20 Plan Fluo NA 0.5) and a total of 100 nuclei and cells were analyzed, for each polymer film. The area, the perimeter, the major and minor axis of nuclei and cells were measured to quantify the variation of the nuclear shape index (NSI), the cell shape index (CSI), the aspect ratio (AR), the cell length (CL), Feret angle and the nuclear positioning. The nuclear positioning is reported as the ratio of the maximum cell length and the maximum cell length protrusion (measured as maximum length from the nucleus).

The coordinate nucleation of F-actin and microtubules in fluorescent images was evaluated using two different Fiji filters on each channel. First, the 8bit images were duplicated and the “north shadow” filter was applied to one. The “convolve” filter and the corresponding color were applied to the duplicated image. The final image is the result of two merged elaborated images (north shadow+convolve colored).

### 4.10. Fluorescence Intensity Quantification

Fluorescence intensity quantification was performed with a custom-made Matlab image analysis code. For each substrate, 100 stained cells were acquired and evaluated. Image enhancement operations were performed on each image before quantification: i) correct non-uniform illumination and ii) background subtraction. First, to correct shading distortion, a Gaussian smoothing filter was applied (imflatfield function implemented in MatLab). Second, to create a background image to be subtracted from the original image, an opening morphological operation was performed on each image, using a disk of 20-pixel diameter (imopen function implemented in Matlab). Finally, each image was thresholded, cells were masked and region properties (integrated density and area) of the masked region of interest (ROI) were calculated.

A fluorescence intensity projection of the cells was then performed on each grayscale image and measured from the relationship:

$$CTCF\;=\;cFID-\left(\frac{Ac}{MAb}*MFB\right)$$

where *CTCF* = corrected total cell fluorescence, *cFID* = cell fluorescence integrated density, *Ac* = area of masked cell, *MAb* = mean area of background (mean of five different ROI) and MFB = mean fluorescence of background (mean of five different ROI).

### 4.11. Field Emission Scanning Electron Microscopy of BM-MSCs on Polymer Films

Stem cell-film interaction was evaluated by FESEM at each time of culture as above. hBM-MSC PBCE, BDG10 and BDG30 squares were rinsed twice with PBS and fixed in 2.5% glutaraldehyde for 30 min at RT then dehydrated by adding progressively more concentrated ethanol (5–100% *v/v*) every 5 min and finally dried by the critical point machine (CPD, Emitech K850, Quorum Technologies, Lewes, UK). Once dried, the samples were gold sputter-coated before examination by FESEM (Supra 25 Zeiss) at an accelerating voltage of 5 kV.

### 4.12. Statistical Analysis

Data analysis was reported as mean ± SD, median and range of intervals (boxplot) and percentage. A post-hoc comparison test was carried out using the one-way ANOVA and Dunn’s Multiple Comparison Test (GraphPad 4.0 Software, San Diego, CA, USA). The *p* < 0.05 was considered statistically significant. Pearson’s bivariate correlation test was evaluated by R—Statistical Computing (https://cran.r-project.org). The *p* < 0.05 was considered statistically significant.

## 5. Conclusions

Collectively, our results provide an innovative polymeric platform, realized with an aliphatic-ring containing polyester (PBCE) processed by the simple solvent cast, whose surface microstructure revealed suitable to drive a surprisingly fast hBM-MSCs fate toward a neuronal-like phenotype in the culture medium, even in the absence of FBS.

## Figures and Tables

**Table 1:** Thermal, and wettability properties of the PBCE-based films. Thermal, X-Ray Diffraction (XRD) and Wettability properties of PBCE*, PBCE, BDG10 and BDG30 polymer films. Thermal properties were analyzed by DSC: melting temperatures (T_m_) and enthalpies (ΔH_m_) were calculated from the first and second heating scans, while glass transition temperature (T_g_) and the specific heat increment (ΔC_p_) were calculated from the second heating scan. Wettability was analyzed by water contact angle (WCA) measurements by using sessile drop method in air. PBCE* measurements were from References n.22 and n.23.

	DSC						XRD	WCA
	I SCAN		II SCAN					
Polymers	T_m_ (°C)	∆H_m_ (Jg^−1^)	T_g_ (°C)	ΔC_p_ (Jg^−1^ °C^−1^)	∆H_m_ (Jg^−1^)	T_m_ (°C)	X_c_ (%)	(°)
PBCE*	166 ± 1	33 ± 1	12 ± 1	0.065 ± 0.002	31 ± 1	167 ± 1	42 ± 3	110 ± 2
**PBCE**	167 ± 1	45 ± 1	15 ± 1	0.082 ± 0.002	31 ± 1	167 ± 1	52 ± 3	100 ± 1
**P(BCE90BDG10)**	155 ± 1	37 ± 1	−2 ± 1	0.091 ± 0.002	29 ± 1	155 ± 1	45 ± 1	95 ± 2
**P(BCE70BDG30)**	120 ± 1	27 ± 1	−17 ± 1	0.263 ± 0.002	21 ± 1	120 ± 1	42 ± 1	86 ± 1

## Supplementary Materials

The following are available online at https://www.mdpi.com/1422-0067/21/23/9274/s1, This file includes Supplementary Figures S1–S9 with their related captions (PDF).

## References

[1] Shafiee A., Atala A. (2017). Tissue Engineering: Toward a New Era of Medicine. Annu. Rev. Med..

[2] Dzobo K., Thomford N.E., Senthebane D.A., Shipanga H., Rowe A., Dandara C., Pillay M., Motaung K.S.C.M. (2018). Advances in Regenerative Medicine and Tissue Engineering: Innovation and Transformation of Medicine. Stem. Cells Int..

[3] Martino S., D’Angelo F., Armentano I., Kenny J.M., Orlacchio A. (2012). Stem cell-biomaterial interactions for regenerative medicine. Biotechnol. Adv..

[4] Downing T.L., Soto J., Morez C., Houssin T., Fritz A., Yuan F., Chu J., Patel S., Schaffer D.V., Li S. (2013). Biophysical regulation of epigenetic state and cell reprogramming. Nat. Mater..

[5] Von Erlach T.C., Bertazzo S., Wozniak M.A., Horejs C.-M., Maynard S.A., Attwood S., Robinson B.K., Autefage H., Kallepitis C., Del Río Hernández A. (2018). Cell-geometry-dependent changes in plasma membrane order direct stem cell signalling and fate. Nat. Mater..

[6] Crowder S.W., Leonardo V., Whittaker T., Papathanasiou P., Stevens M.M. (2016). Material Cues as Potent Regulators of Epigenetics and Stem Cell Function. Cell Stem. Cell.

[7] Di Cio S., Gautrot J.E. (2016). Cell sensing of physical properties at the nanoscale: Mechanisms and control of cell adhesion and phenotype. Acta Biomater..

[8] Theocharis A.D., Skandalis S.S., Gialeli C., Karamanos N.K. (2016). Extracellular matrix structure. Adv. Drug Deliv. Rev..

[9] Hussey G.S., Dziki J.L., Badylak S.F. (2018). Extracellular matrix-based materials for regenerative medicine. Nat. Rev. Mater..

[10] Ingber D.E., Tensegrity I. (2003). Cell structure and hierarchical systems biology. J. Cell Sci..

[11] Ingber D.E. (2003). Tensegrity II. How structural networks influence cellular information processing networks. J. Cell. Sci..

[12] Schiffhauer E.S., Robinson D.N. (2017). Mechanochemical Signaling Directs Cell-Shape Change. Biophys. J..

[13] Hiew V.V., Simat S.F.B., Teoh P.L. (2018). The Advancement of Biomaterials in Regulating Stem Cell Fate. Stem. Cell Rev. Rep..

[14] Argentati C., Morena F., Tortorella I., Bazzucchi M., Porcellati S., Emiliani C., Martino S. (2019). Insight into Mechanobiology: How Stem Cells Feel Mechanical Forces and Orchestrate Biological Functions. Int. J. Mol. Sci..

[15] Chacón-Martínez C.A., Koester J., Wickström S.A. (2018). Signaling in the stem cell niche: Regulating cell fate, function and plasticity. Development.

[16] Gattazzo F., Urciuolo A., Bonaldo P. (2014). Extracellular matrix: A dynamic microenvironment for stem cell niche. Biochim. Biophys. Acta.

[17] Morena F., Armentano I., Montanucci P., Argentati C., Fortunati E., Montesano S., Bicchi I., Pescara T., Pennoni I., Mattioli S. (2017). Design of a nanocomposite substrate inducing adult stem cell assembly and progression toward an Epiblast-like or Primitive Endoderm-like phenotype via mechanotransduction. Biomaterials.

[18] González-Díaz E.C., Varghese S. (2016). Hydrogels as Extracellular Matrix Analogs. Gels.

[19] Burdick J.A., Prestwich G.D. (2011). Hyaluronic acid hydrogels for biomedical applications. Adv. Mater. Weinheim.

[20] Ayala R., Zhang C., Yang D., Hwang Y., Aung A., Shroff S.S., Arce F.T., Lal R., Arya G., Varghese S. (2011). Engineering the cell-material interface for controlling stem cell adhesion, migration, and differentiation. Biomaterials.

[21] D’Angelo F., Armentano I., Cacciotti I., Tiribuzi R., Quattrocelli M., Del Gaudio C., Fortunati E., Saino E., Caraffa A., Cerulli G.G. (2012). Tuning multi/pluri-potent stem cell fate by electrospun poly(L-lactic acid)-calcium-deficient hydroxyapatite nanocomposite mats. Biomacromolecules.

[22] Argentati C., Morena F., Montanucci P., Rallini M., Basta G., Calabrese N., Calafiore R., Cordellini M., Emiliani C., Armentano I. (2018). Surface Hydrophilicity of Poly(l-Lactide) Acid Polymer Film Changes the Human Adult Adipose Stem Cell Architecture. Polymers.

[23] Murphy W.L., McDevitt T.C., Engler A.J. (2014). Materials as stem cell regulators. Nat. Mater..

[24] Ahmadi S.M., Kumar R., Borisov E.V., Petrov R., Leeflang S., Li Y., Tümer N., Huizenga R., Ayas C., Zadpoor A.A. (2019). From microstructural design to surface engineering: A tailored approach for improving fatigue life of additively manufactured meta-biomaterials. Acta Biomater..

[25] Mukherjee D.P., Smith D.F., Rogers S.H., Emmanual J.E., Jadin K.D., Hayes B.K. (2009). Effect of 3D-microstructure of bioabsorbable PGA:TMC scaffolds on the growth of chondrogenic cells. J. Biomed. Mater. Res. Part B Appl. Biomater..

[26] Lee B.L.-P., Tang Z., Wang A., Huang F., Yan Z., Wang D., Chu J.S., Dixit N., Yang L., Li S. (2013). Synovial stem cells and their responses to the porosity of microfibrous scaffold. Acta Biomater..

[27] Lin X., Shi Y., Cao Y., Liu W. (2016). Recent progress in stem cell differentiation directed by material and mechanical cues. Biomed. Mater..

[28] Wen J.H., Vincent L.G., Fuhrmann A., Choi Y.S., Hribar K.C., Taylor-Weiner H., Chen S., Engler A.J. (2014). Interplay of matrix stiffness and protein tethering in stem cell differentiation. Nat. Mater..

[29] Gupta M., Doss B.L., Kocgozlu L., Pan M., Mège R.-M., Callan-Jones A., Voituriez R., Ladoux B. (2019). Cell shape and substrate stiffness drive actin-based cell polarity. Phys. Rev. E.

[30] Ferrari M., Cirisano F., Morán M.C. (2019). Mammalian Cell Behavior on Hydrophobic Substrates: Influence of Surface Properties. Colloids Interfaces.

[31] Song W., Veiga D.D., Custódio C.A., Mano J.F. (2009). Bioinspired Degradable Substrates with Extreme Wettability Properties. Adv. Mater..

[32] Van Wachem P.B., Beugeling T., Feijen J., Bantjes A., Detmers J.P., van Aken W.G. (1985). Interaction of cultured human endothelial cells with polymeric surfaces of different wettabilities. Biomaterials.

[33] Ahn H.H., Lee I.W., Lee H.B., Kim M.S. (2014). Cellular behavior of human adipose-derived stem cells on wettable gradient polyethylene surfaces. Int. J. Mol. Sci..

[34] Chen H., Gigli M., Gualandi C., Truckenmüller R., van Blitterswijk C., Lotti N., Munari A., Focarete M.L., Moroni L. (2016). Tailoring chemical and physical properties of fibrous scaffolds from block copolyesters containing ether and thio-ether linkages for skeletal differentiation of human mesenchymal stromal cells. Biomaterials.

[35] Cristofaro F., Gigli M., Bloise N., Chen H., Bruni G., Munari A., Moroni L., Lotti N., Visai L. (2018). Influence of the nanofiber chemistry and orientation of biodegradable poly(butylene succinate)-based scaffolds on osteoblast differentiation for bone tissue regeneration. Nanoscale.

[36] Gualandi C., Soccio M., Saino E., Focarete M.L., Lotti N., Munari A., Moroni L., Visai L. (2012). Easily synthesized novel biodegradable copolyesters with adjustable properties for biomedical applications. Soft Matter.

[37] Gigli M., Lotti N., Vercellino M., Visai L., Munari A. (2014). Novel ether-linkages containing aliphatic copolyesters of poly(butylene 1,4-cyclohexanedicarboxylate) as promising candidates for biomedical applications. Mater. Sci. Eng. C Mater. Biol. Appl..

[38] Bloise N., Berardi E., Gualandi C., Zaghi E., Gigli M., Duelen R., Ceccarelli G., Cortesi E.E., Costamagna D., Bruni G. (2018). Ether-Oxygen Containing Electrospun Microfibrous and Sub-Microfibrous Scaffolds Based on Poly(butylene 1,4-cyclohexanedicarboxylate) for Skeletal Muscle Tissue Engineering. Int. J. Mol. Sci..

[39] Fortunati E., D’Angelo F., Martino S., Orlacchio A., Kenny J.M., Armentano I. (2011). Carbon nanotubes and silver nanoparticles for multifunctional conductive biopolymer composites. Carbon.

[40] Lizundia E., Sarasua J.R., D’Angelo F., Orlacchio A., Martino S., Kenny J.M., Armentano I. (2012). Biocompatible poly(L-lactide)/MWCNT nanocomposites: Morphological characterization, electrical properties, and stem cell interaction. Macromol. Biosci..

[41] Armentano I., Ciapetti G., Pennacchi M., Dottori M., Devescovi V., Granchi D., Baldini N., Olalde B., Jurado M.J., Alava J.I.M. (2009). Role of PLLA plasma surface modification in the interaction with human marrow stromal cells. J. Appl. Polym. Sci..

[42] D’Angelo F., Armentano I., Mattioli S., Crispoltoni L., Tiribuzi R., Cerulli G.G., Palmerini C.A., Kenny J.M., Martino S., Orlacchio A. (2010). Micropatterned hydrogenated amorphous carbon guides mesenchymal stem cells towards neuronal differentiation. Eur. Cell Mater..

[43] Martino S., D’Angelo F., Armentano I., Tiribuzi R., Pennacchi M., Dottori M., Mattioli S., Caraffa A., Cerulli G.G., Kenny J.M. (2009). Hydrogenated amorphous carbon nanopatterned film designs drive human bone marrow mesenchymal stem cell cytoskeleton architecture. Tissue Eng. Part A.

[44] Morena F., Argentati C., Calzoni E., Cordellini M., Emiliani C., D’Angelo F., Martino S. (2016). Ex-Vivo Tissues Engineering Modeling for Reconstructive Surgery Using Human Adult Adipose Stem Cells and Polymeric Nanostructured Matrix. Nanomaterials.

[45] Fortunati E., Gigli M., Luzi F., Lotti N., Munari A., Gazzano M., Armentano I., Kenny J.M. Poly(Butylene Cyclohexanedicarboxylate/Diglycolate) Random Copolymers Reinforced with SWCNTs for Multifunctional Conductive Biopolymer Composites. https://www.ingentaconnect.com/content/doaj/1788618x/2016/00000010/00000002/art00003;jsessionid=37b3ejhn0mt93.x-ic-live-02.

[46] Pittenger M.F., Discher D.E., Péault B.M., Phinney D.G., Hare J.M., Caplan A.I. (2019). Mesenchymal stem cell perspective: Cell biology to clinical progress. NPJ Regen. Med..

[47] Lin H., Sohn J., Shen H., Langhans M.T., Tuan R.S. (2019). Bone marrow mesenchymal stem cells: Aging and tissue engineering applications to enhance bone healing. Biomaterials.

[48] Zheng Y., Huang C., Liu F., Lin H., Yang X., Zhang Z. (2017). Comparison of the neuronal differentiation abilities of bone marrow‑derived and adipose tissue‑derived mesenchymal stem cells. Mol. Med. Rep..

[49] Woodbury D., Schwarz E.J., Prockop D.J., Black I.B. (2000). Adult rat and human bone marrow stromal cells differentiate into neurons. J. Neurosci. Res..

[50] Wang Y., Chen X., Cao W., Shi Y. (2014). Plasticity of mesenchymal stem cells in immunomodulation: Pathological and therapeutic implications. Nat. Immunol..

[51] Gao F., Chiu S.M., Motan D.a.L., Zhang Z., Chen L., Ji H.-L., Tse H.-F., Fu Q.-L., Lian Q. (2016). Mesenchymal stem cells and immunomodulation: Current status and future prospects. Cell Death Dis..

[52] Andrzejewska A., Lukomska B., Janowski M. (2019). Concise Review: Mesenchymal Stem Cells: From Roots to Boost. Stem. Cells.

[53] Rushing G., Ihrie R.A. (2016). Neural stem cell heterogeneity through time and space in the ventricular-subventricular zone. Front. Biol..

[54] Xie L., Zeng X., Hu J., Chen Q. (2015). Characterization of Nestin, a Selective Marker for Bone Marrow Derived Mesenchymal Stem Cells. Stem. Cells Int..

[55] Yang Y., Pang D., Hu C., Lv Y., He T., An Y., Tang Z., Deng Z. (2015). Nestin Positive Bone Marrow Derived Cells Responded to Injury Mobilize into Peripheral Circulation and Participate in Skin Defect Healing. PLoS ONE.

[56] Lindsay S.L., Barnett S.C. (2017). Are nestin-positive mesenchymal stromal cells a better source of cells for CNS repair?. Neurochem. Int..

[57] Hennrich M.L., Romanov N., Horn P., Jaeger S., Eckstein V., Steeples V., Ye F., Ding X., Poisa-Beiro L., Lai M.C. (2018). Cell-specific proteome analyses of human bone marrow reveal molecular features of age-dependent functional decline. Nat. Commun..

[58] Armentano I., Fortunati E., Gigli M., Luzi F., Trotta R., Bicchi I., Soccio M., Lotti N., Munari A., Martino S. (2016). Effect of SWCNT introduction in random copolymers on material properties and fibroblast long term culture stability. Polym. Degrad. Stab..

[59] Ramkumar N., Baum B. (2016). Coupling changes in cell shape to chromosome segregation. Nat. Rev. Mol. Cell Biol..

[60] Prasad A., Alizadeh E. (2019). Cell Form and Function: Interpreting and Controlling the Shape of Adherent Cells. Trends Biotechnol..

[61] Charrier E.E., Pogoda K., Wells R.G., Janmey P.A. (2018). Control of cell morphology and differentiation by substrates with independently tunable elasticity and viscous dissipation. Nat. Commun..

[62] Ornaghi F., Sala D., Tedeschi F., Maffia M.C., Bazzucchi M., Morena F., Valsecchi M., Aureli M., Martino S., Gritti A. (2020). Novel bicistronic lentiviral vectors correct β-Hexosaminidase deficiency in neural and hematopoietic stem cells and progeny: Implications for in vivo and ex vivo gene therapy of GM2 gangliosidosis. Neurobiol. Dis..

[63] Martino S., di Girolamo I., Cavazzin C., Tiribuzi R., Galli R., Rivaroli A., Valsecchi M., Sandhoff K., Sonnino S., Vescovi A. (2009). Neural precursor cell cultures from GM2 gangliosidosis animal models recapitulate the biochemical and molecular hallmarks of the brain pathology. J. Neurochem..

[64] Meneghini V., Lattanzi A., Tiradani L., Bravo G., Morena F., Sanvito F., Calabria A., Bringas J., Fisher-Perkins J.M., Dufour J.P. (2016). Pervasive supply of therapeutic lysosomal enzymes in the CNS of normal and Krabbe-affected non-human primates by intracerebral lentiviral gene therapy. EMBO Mol. Med..

[65] Meneghini V., Frati G., Sala D., De Cicco S., Luciani M., Cavazzin C., Paulis M., Mentzen W., Morena F., Giannelli S. (2017). Generation of Human Induced Pluripotent Stem Cell-Derived Bona Fide Neural Stem Cells for Ex Vivo Gene Therapy of Metachromatic Leukodystrophy. Stem. Cells Transl. Med..

[66] Lin G.-L., Wang H., Dai J., Li X., Guan M., Ding Q., Wang H.-X., Fang H. (2018). Upregulation of UBAP2L in Bone Marrow Mesenchymal Stem Cells Promotes Functional Recovery in Rats with Spinal Cord Injury. Curr. Med. Sci..

[67] Cheng O., Tian X., Luo Y., Mai S., Yang Y., Kuang S., Chen Q., Ma J., Chen B., Li R. (2018). Liver X receptors agonist promotes differentiation of rat bone marrow derived mesenchymal stem cells into dopaminergic neuron-like cells. Oncotarget.

[68] Long Q., Luo Q., Wang K., Bates A., Shetty A.K. (2017). Mash1-dependent Notch Signaling Pathway Regulates GABAergic Neuron-Like Differentiation from Bone Marrow-Derived Mesenchymal Stem Cells. Aging Dis..

[69] Heo J.S., Choi S.-M., Kim H.O., Kim E.H., You J., Park T., Kim E., Kim H.-S. (2013). Neural transdifferentiation of human bone marrow mesenchymal stem cells on hydrophobic polymer-modified surface and therapeutic effects in an animal model of ischemic stroke. Neuroscience.

[70] Poudineh M., Wang Z., Labib M., Ahmadi M., Zhang L., Das J., Ahmed S., Angers S., Kelley S.O. (2018). Three-Dimensional Nanostructured Architectures Enable Efficient Neural Differentiation of Mesenchymal Stem Cells via Mechanotransduction. Nano Lett..

[71] Wu G.-H., Shi H.-J., Che M.-T., Huang M.-Y., Wei Q.-S., Feng B., Ma Y.-H., Wang L.-J., Jiang B., Wang Y.-Q. (2018). Recovery of paralyzed limb motor function in canine with complete spinal cord injury following implantation of MSC-derived neural network tissue. Biomaterials.

[72] Metavarayuth K., Sitasuwan P., Zhao X., Lin Y., Wang Q. (2016). Influence of Surface Topographical Cues on the Differentiation of Mesenchymal Stem Cells in Vitro. ACS Biomater. Sci. Eng..

[73] Wang Z., Zhang L., Labib M., Chen H., Wei M., Poudineh M., Green B.J., Duong B., Das J., Ahmed S. (2019). Peptide-Functionalized Nanostructured Microarchitectures Enable Rapid Mechanotransductive Differentiation. ACS Appl. Mater. Interfaces.

[74] Bradford M.M. (1976). A rapid and sensitive method for the quantitation of microgram quantities of protein utilizing the principle of protein-dye binding. Anal. Biochem..

[75] D’Aquino R., Tirino V., Desiderio V., Studer M., De Angelis G.C., Laino L., De Rosa A., Di Nucci D., Martino S., Paino F. (2011). Human neural crest-derived postnatal cells exhibit remarkable embryonic attributes either in vitro or in vivo. Eur. Cell Mater..

