# Supplementary material for: Unpatterned Bioactive Poly(Butylene 1,4-Cyclohexanedicarboxylate)-Based Film Fast Induced Neuronal-Like Differentiation of Human Bone Marrow-Mesenchymal Stem Cells
Source: Int J Mol Sci. 2020 Dec 4;21(23):9274. doi: 10.3390/ijms21239274 (PMC7729499; doi:10.3390/ijms21239274)
Supplement: Supplementary file 1 [file ijms-21-09274-s001.pdf]

Supporting Information

Unpatterned bioactive poly(butylene 1,4-cyclohexanedicarboxylate)-based films fast inducing neuronal reprogramming of human bone marrow-mesenchymal stem cells

Francesco Morena[1*], Chiara Argentati[1*], Michelina Soccio[2*], Ilaria Bicchi[1], Francesca Luzi[3], Luigi Torre[3], Andrea Munari[2], Carla Emiliani[1,4], Matteo Gigli[5], Nadia Lotti[2,§], Ilaria Armentano[6,§], Sabata Martino[1,4,§]

[1]Department of Chemistry, Biology and Biotechnology, University of Perugia, Perugia, Italy

[2]Department of Civil, Chemical, Environmental, and Materials Engineering–DICAM, University of Bologna, Bologna, Italy

[3]Civil and Environmental Engineering Department, UdR INSTM, University of Perugia, Terni, Italy

[4]CEMIN, University of Perugia, Perugia, Italy

[5]Department of Molecular Sciences and Nanosystems, Ca'Foscari University of Venice, Venezia Mestre, Italy

[6]Department of Economics, Engineering, Society and Business Organization (DEIM), University of Tuscia, Viterbo, Italy

Supporting information file includes Supplementary Figures S1-S9 with related captions.

Supplementary Figures

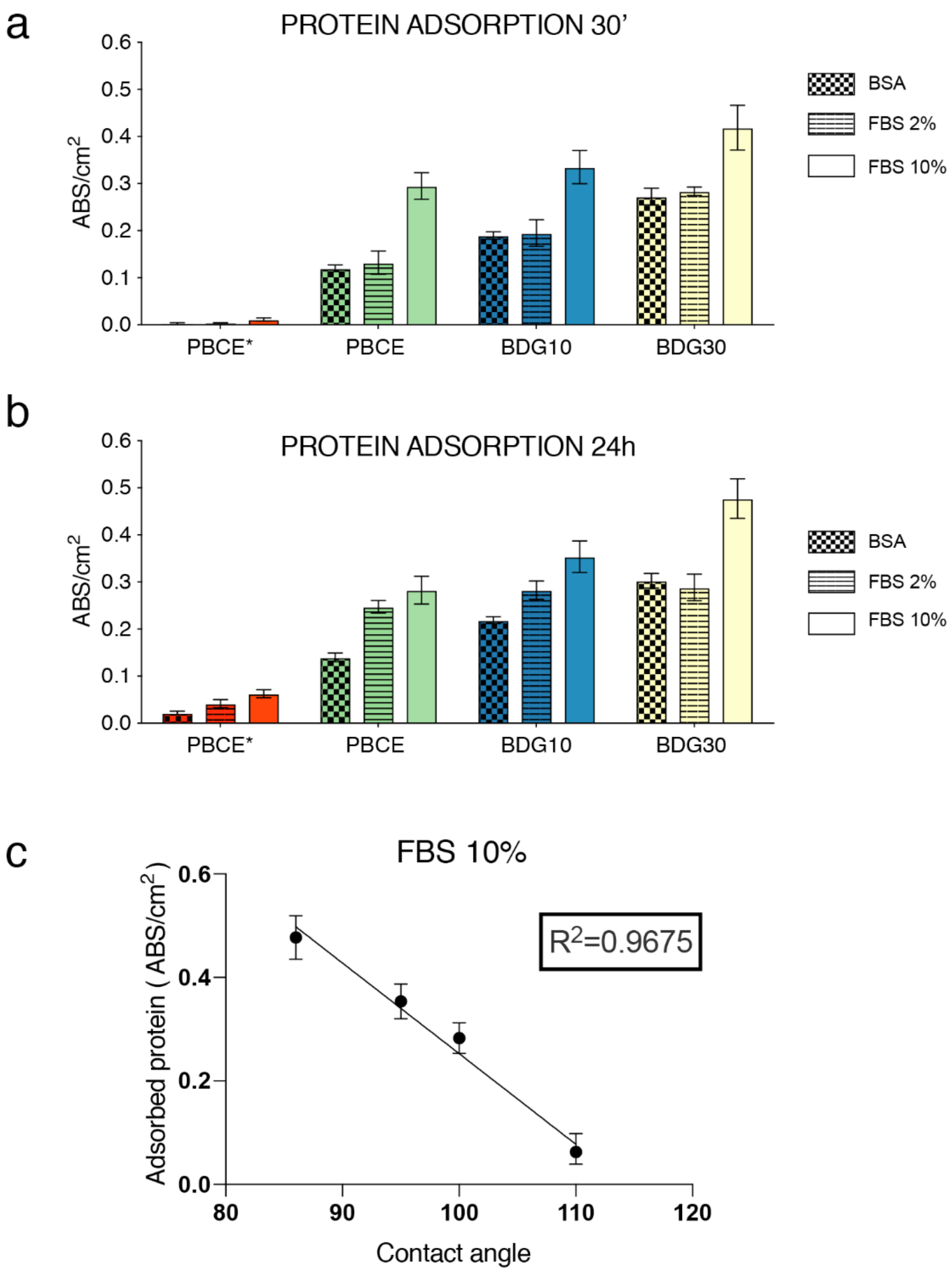

Figure S1. Protein adsorption on PBCE*, PBCE, BDG10 and BDG30. Level of BSA, FBS 2% and FBS 10% after the protein adsorption assay at 30 min (a) and 24 h (b) time points on PBCE*, PBCE, BDG10 and BDG30, respectively. Control experiments were carried out by incubating 1cm^2 square of PBCE, BDG10 and BDG30 with H_20d. (c) Linear regression analysis of the relationship between polymer films contact angle values and adsorbed proteins. The results are expressed as mean±SD of three independent experiments.

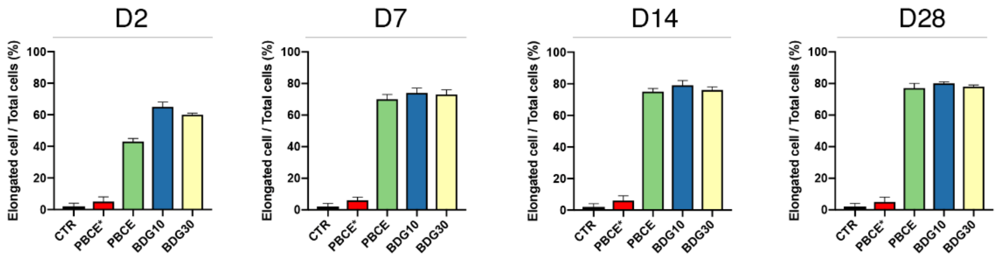

Figure S2. Percentage of elongated and polarized hBM-MSCs on PBCE*, PBCE, BDG10 and BDG30. The percentage of elongate hBM-MSCs on polymer films is calculated with respect to total stem cells. Elongated stem cells are considered all cells that were longer than stem cells on CTR (TCP/GC), and polarized. The results are expressed as mean±SD of three independent experiments.

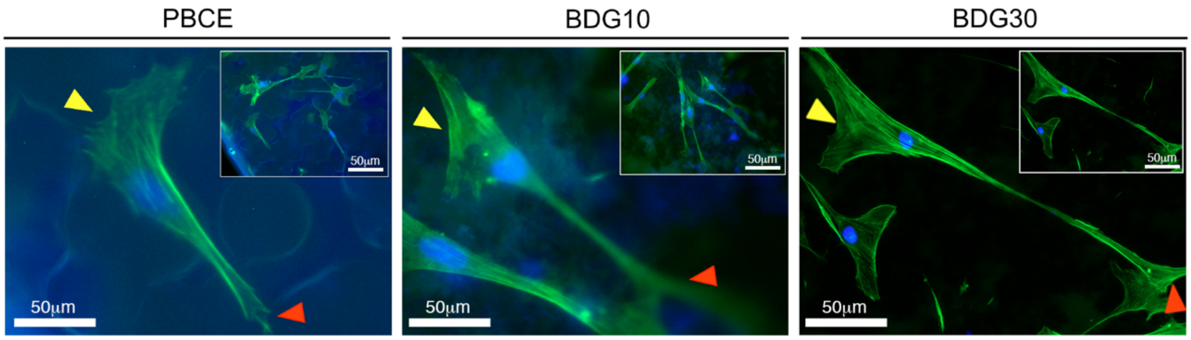

Figure S3. High magnification of elongated stem cells on PBCE-films. Fluorescence images of hBM-MSC nuclei (DAPI, blue) and F-actin (FITC-phalloidin, green) revealed the stem elongation and polarization on PBCE-films. The cell body enlargement (yellow arrow) and the cell protrusion (red arrow) are indicated.

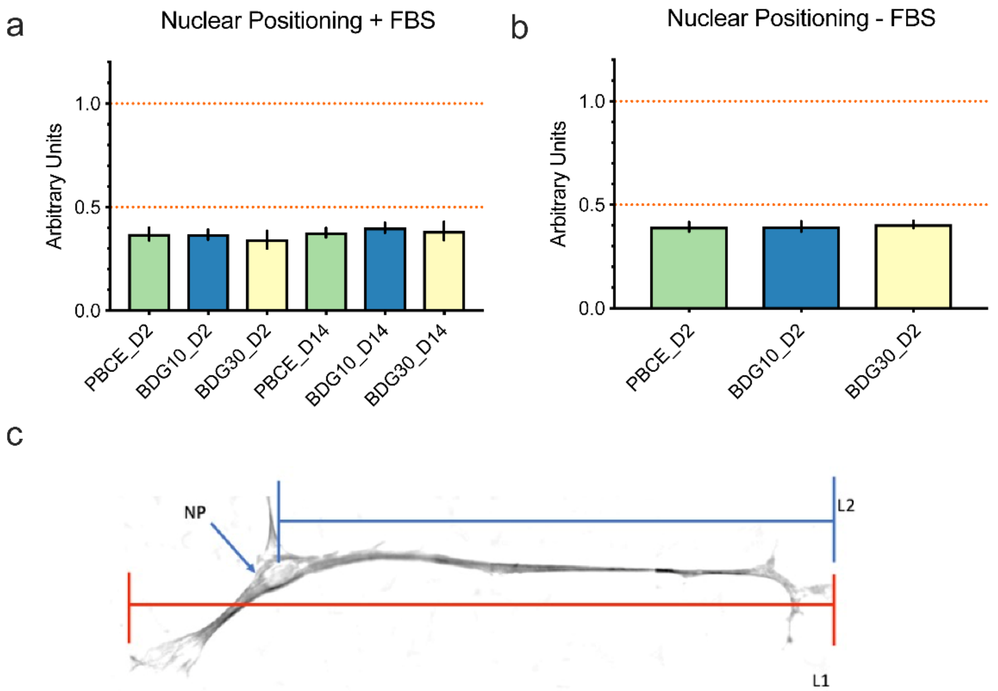

Figure S4. Nuclear positioning in hBM-MSCs on PBCE-films. (a,b) The positioning of nuclei in elongated polarized stem cells on polymer films was measured as % of the displacement with respect to total cell length. (a) Nuclear positioning on D2 and on D14. (b) Nuclear positioning on D2 in stem cells grown in the absence of FBS. (c) Nuclear positioning measurement. L1: Cell length measured from GC-like protrusion and along medial axis of cell; L2: Cell length protrusion measured as maximum length from nucleus; NP: Nuclear positioning measured as ratio of L2/L1.

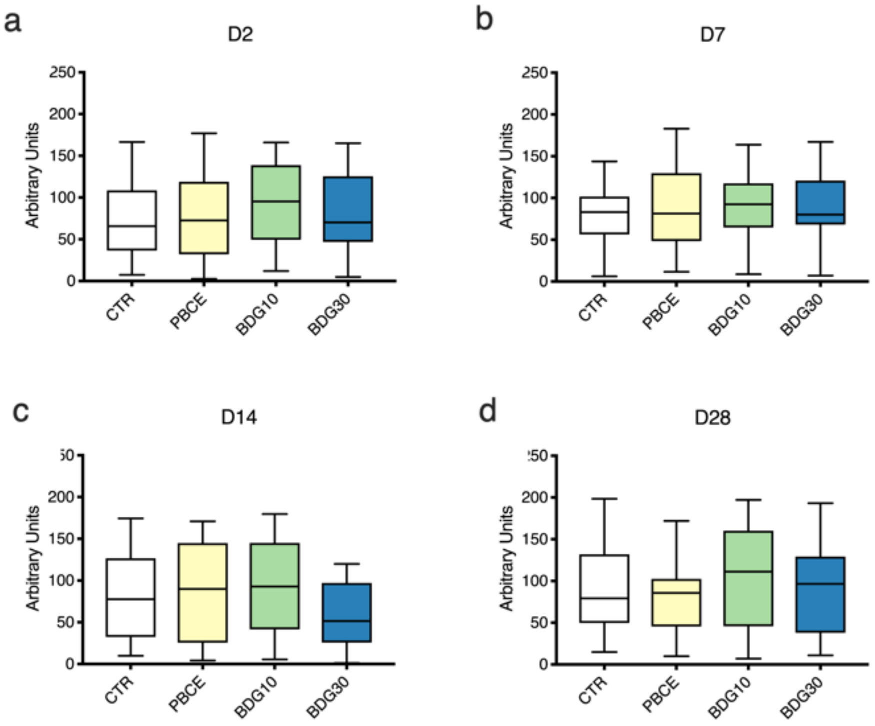

Figure S5. Feret angle measures in hBM-MSCs on PBCE-films. Feret Angle, that measures the directional angle of the Feret diameter with respect to the horizontal axis of the image, was determined as described in materials and methods section. Measures were conducted on D2 (a), D7 (b), D14 (c) and D28 (d). The results are expressed as mean of three independent experiments.

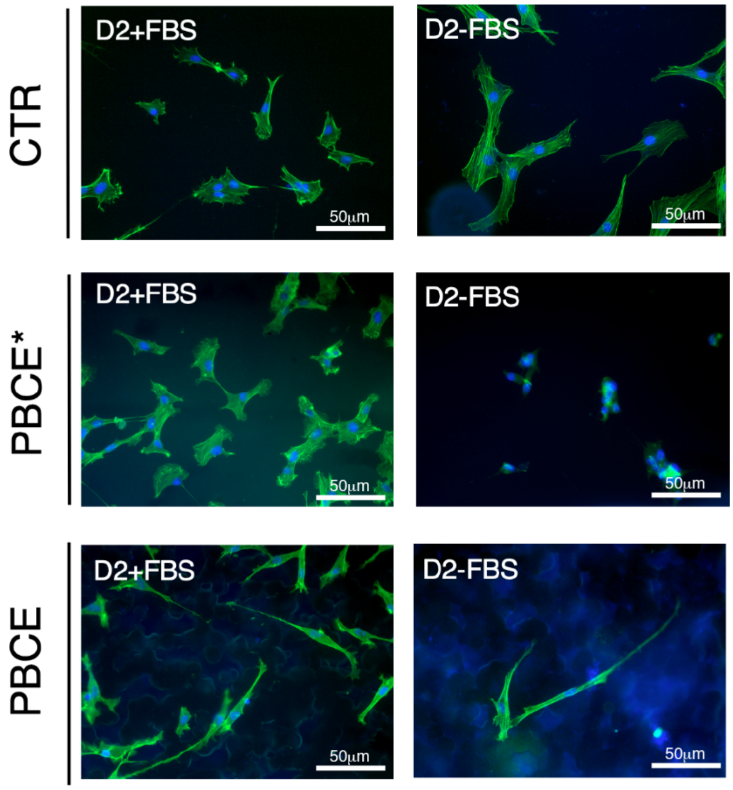

Figure S6. Culture of hBM-MSCs on CTR, PBCE* and on PBCE in the presence and absence of FBS on D2. Representative fluorescence images of hBM-MSC nuclei (DAPI, blue) and F-actin (FITC-phalloidin, green) revealed the stem cell canonical fibroblast-like morphology on PBCE* in the presence of FBS and on CTR culture in both presence/absence of FBS, and showed the change of the cell shape on PBCE in both culture condition. Few cells were observed on PBCE* in the absence of FBS. Scale bar 50 μm.

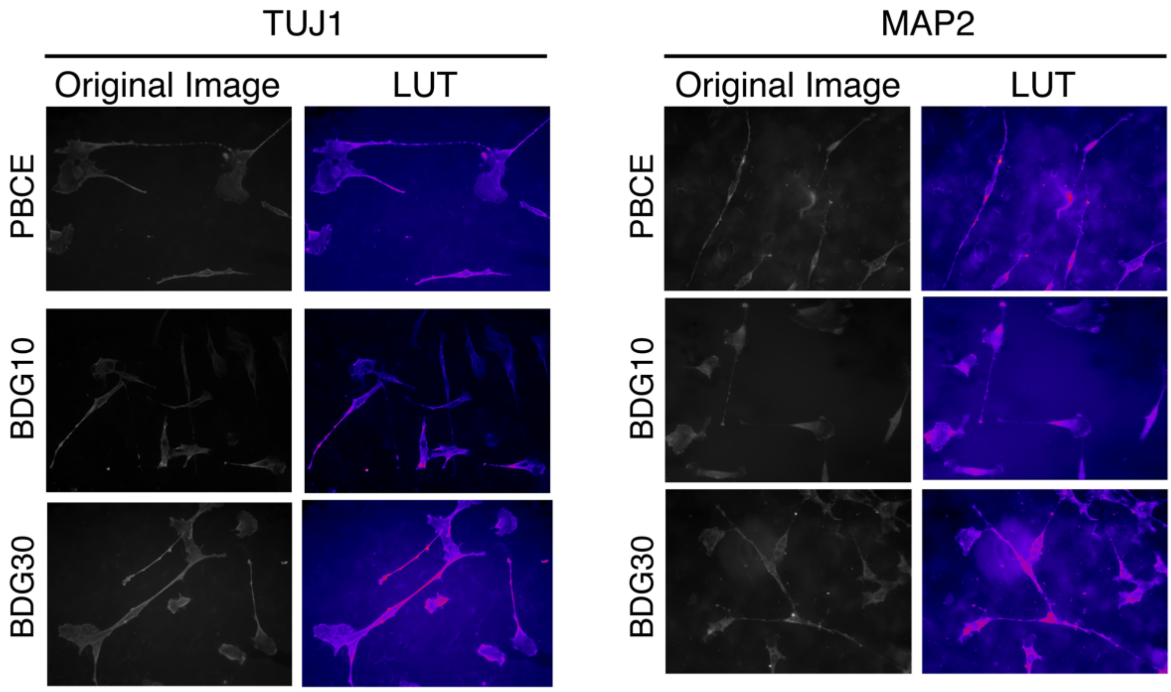

Figure S7. Gray scales levels of images in Figure 7. Visual of slight differences in gray values can be facilitated with the use of color look-up tables (LUTs). This allows displaying gray levels according to a set map of colors rather than intensity.

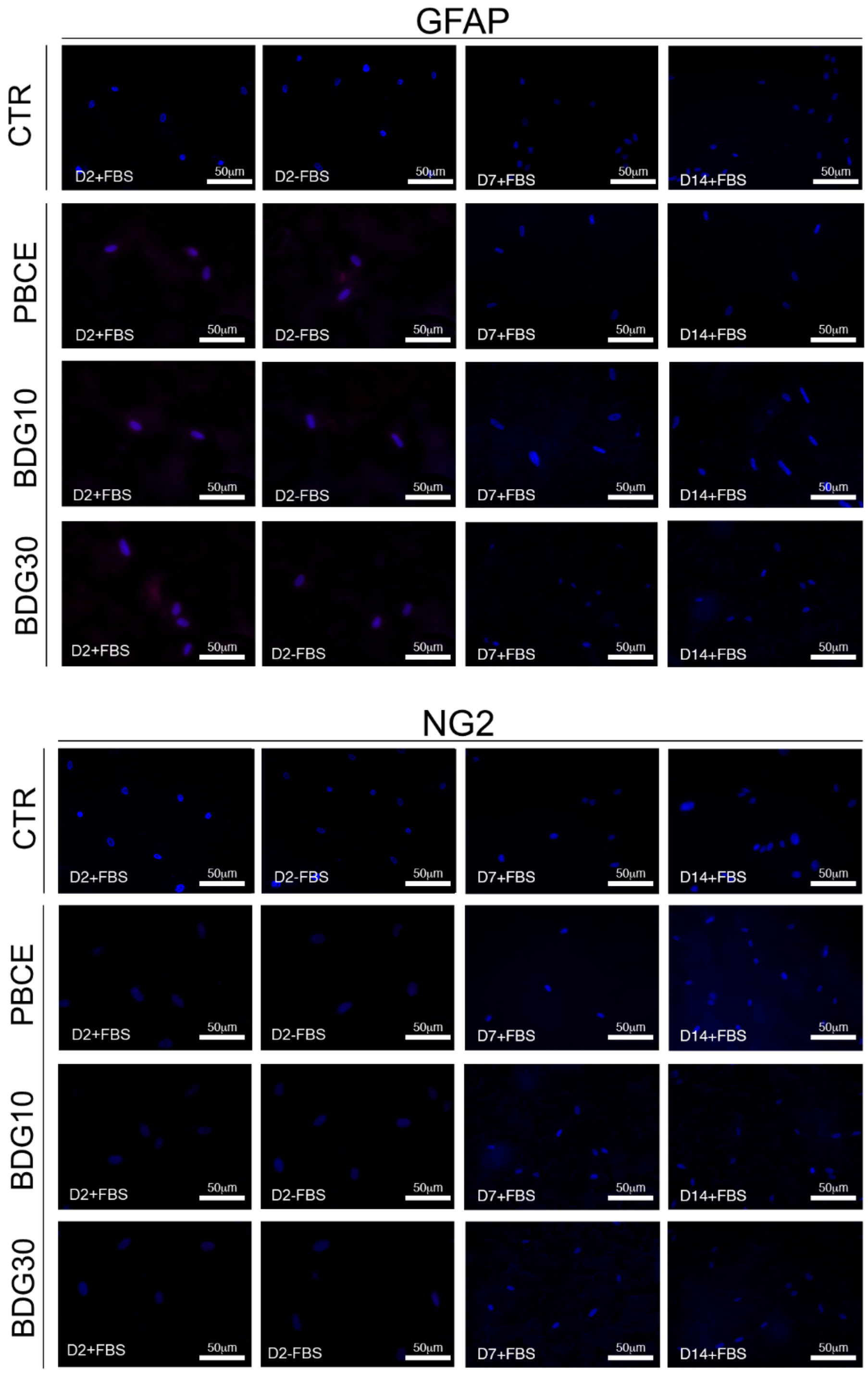

Figure S8. Expression of GFAP and NG2 in hBM-MSCs grown on PBCE, BDG10 and BDG30. Representative fluorescence images of hBM-MSC nuclei (DAPI, blue), GFAP (human anti-GFAP antibody, red), and NG2 (human anti-NG2 antibody, red), revealed the absence of both glial markers in cells on CTR cultures and on polymer films, both in the presence (+) and absence (-) of FBS. Scale bar 50 μm.

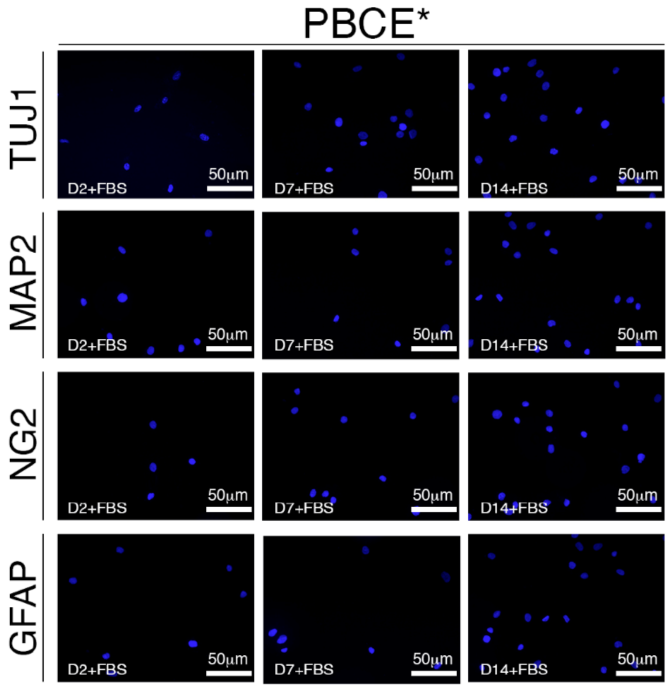

Figure S9. Expression of TUJ1, MAP2, GFAP and NG2 in hBM-MSCs grown on PBCE*. Representative fluorescence images of hBM-MSC nuclei (DAPI, blue), TUJ1 (human anti-TJU1 antibody, green), and MAP2 (human anti-MAP2 antibody, red), GFAP (human anti-GFAP antibody, red), and NG2 (human anti-NG2 antibody, red), revealed the absence of neuronal and glial markers in cells on PBCE*, both in the presence of FBS. Scale bar 50 µm.
